# Process Optimization and Flavor Analysis of *Lespedeza juncea* Tea Based on HS-SPME-GC-O-MS, LC-MS, and Sensory Evaluation

**DOI:** 10.3390/foods15061066

**Published:** 2026-03-18

**Authors:** Hui Fu, Yuning Liu, Pengcheng Qiu, Ying Ying, Yiying Huang, Runhang Li, Qiang Yin, Yuanfa Meng, Zhihui Zhao, Xiaowei Jiang, Hongxin Wu

**Affiliations:** 1 Institute of Grassland Research, Northern Agriculture and Livestock Husbandry Technical Innovation Center, Chinese Academy of Agricultural Sciences, Hohhot 010010, China; 15148049294@163.com (H.F.); yingying@caas.cn (Y.Y.); huangyiyingnm@163.com (Y.H.); lirunhang@caas.cn (R.L.); yinqiang0477@126.com (Q.Y.); nymyf1990@163.com (Y.M.); zhaozhihui@caas.cn (Z.Z.); jiangxiaowei@caas.cn (X.J.); 2 Ordos Agricultural and Livestock Products and Safety Center, Ordos 017200, China; qpc1548@126.com; 3 Key Field Scientific Observation and Experiment Station for the Ecological Environment of Sandy Grasslands in Ordos, Ministry of Agriculture and Rural Affairs, Ordos 014300, China

**Keywords:** *Lespedeza juncea* tea, antioxidant activity, HS-SPME-GC-O-MS, LC-MS, sensory evaluation

## Abstract

Based on ancient records of “Marching tea” and the flavonoid-rich properties of *Lespedeza juncea*, this study optimized green tea processing parameters for *Lespedeza juncea* tea. By adjusting leaf loading, roasting temperature, and time and employing precise methods such as gas chromatography–olfactory–mass spectrometry (GC-O-MS), liquid chromatography–mass spectrometry (LC-MS), and electronic tongues to determine key indicators, including metabolites, volatile compounds, and flavor profiles, the optimal processing conditions were determined through comprehensive analysis as 150 g, 200 °C, and 180 s. These conditions resulted in superior sensory quality (score: 90.9), the highest flavonoid content (4.11%), strong antioxidant activity (DPPH: 66.0% at 20 mg/mL; ABTS: >95% at 2.5 mg/mL), and key aroma compounds 1-octen-3-ol and *β*-ionone. This work revives an ancient tea tradition and provides a scientific basis for developing novel functional teas from grassland resources.

## 1. Introduction

*Lespedeza juncea* belongs to the *Fabaceae* family, the *Papilionoideae* subfamily, and the *Lespedeza* genus. This perennial subshrub has narrow, elongated leaves and racemose inflorescences. It thrives in mountainous shrublands and on dry, rocky slopes below 1500 m in elevation. It is primarily distributed across Inner Mongolia, Northeast China, and Shaanxi Province. It exhibits notable cold tolerance, drought resistance, and adaptability to nutrient-poor soils [1,2,3]. *Lespedeza juncea* has traditionally been used as a folk herbal medicine. It dates back to the Ming Dynasty, when it was valued for its detoxifying properties and ability to stop diarrhea, promote urination, and stop bleeding [4,5]. The genus *Lespedeza* is documented in the ancient text “Herbal for Relief of Famines”, which states that “*Lespedeza*, commonly known as ‘Marching tea’, grows in marshy areas. There are two varieties distinguished by leaf size: The large-leaved variety resembles black bean leaf (such as: *Lespedeza bicolor* Turcz., *Lespedeza* thunbergii subsp. Formosa (Vogel) H. Ohashi, *Lespedeza davurica* auct. non (Laxm.) Schindl.: V. N. Vassil.), while the small-leaved variety resembles Achillea (such as: *Lespedeza juncea*)”. The text also details how to make “Marching tea”: “The fresh leaves are picked, steamed and dried to make tea, which can be drunk after boiling.” This reflects *Lespedeza*’s long history of being used for tea. However, this tradition has not been well preserved. Therefore, it is crucial to explore the differences in the main chemical constituents and flavor characteristics of *Lespedeza juncea* tea using both traditional methods and modern tea processing techniques.

Tea can be categorized based on the degree of fermentation: fully fermented, semi-fermented, or unfermented [6]. There are six major types of tea based on different tea-making processes: white tea, green tea, black tea, oolong tea, yellow tea, and Pu’er tea. Compared to other types of tea, green tea is a non-fermented variety; high temperatures can rapidly inactivate enzymes, thus stopping enzymatic reactions and preserving their natural chemical properties [7,8]. Additionally, processes such as rolling and drying enhance the tea’s aroma [8]. Wang et al. produced tea from strawberry leaves by using green tea processing techniques on the leaves, resulting in a clear tea liquor with a sweet aftertaste [9]. Zhang et al. used green tea processing methods to create tea from sea buckthorn leaves, which has a low caffeine content and is thus suitable for those of all ages to drink [10]. Tang et al. produced tea using raspberry leaves as the raw material. This tea exhibited aroma compounds identical to those found in commercially available green tea [11]. Li et al. investigated the effects of different fixation methods on *Lespedeza* tea quality using sensory evaluation but did not conduct in-depth research [2]. Therefore, the raw materials and tea-making process are important factors affecting tea quality [12].

In recent years, developing specialty tea products from non-tea plants using tea processing technology has become a popular research topic. This approach offers a new way to utilize plants for high-value products and helps to meet people’s demand for diverse tea products. Therefore, producing green tea from *Lespedeza juncea* maximizes the retention of its functional components. Using modern chemical composition analysis and sensory evaluation methods, this study provides a basis for recreating “Marching tea” and contributes positively to the comprehensive development and utilization of *Lespedeza* resources.

## 2. Materials and Methods

### 2.1. Chemical Reagents and Instruments

All chemical reagents and solvents were purchased from commercial sources (Sigma-Aldrich (St. Louis, MO, USA), Macklin (Shanghai, China), and Yuanye Inc. (Shanghai, China)) and were used without further purification. Deionized water was prepared using laboratory water purification equipment (CASCADA, Avidity Science, Jiaxing, China). A hand-roasted tea pan (X21) was purchased from Jinrunxing (Shenzhen, China). Colorimetric determinations were performed using a UV–visible spectrophotometer (UV-1780, Shimadzu, Kyoto, Japan). Taste measurements were conducted using an Insent 402B Plus-EX electronic tongue (Insent, Atsugi, Japan). Gas chromatography–mass spectrometry (GC-MS) analysis was performed on the Shimadzu GCMS-TQ8040 NX (Shimadzu, Kyoto, Japan). Liquid chromatography–mass spectrometry (LC-MS) was performed on a Thermo Fisher Vanquish UHPLC and Thermo Fisher Q Exactive^TM^ HF-X (Thermo Fisher, Waltham, MA, USA).

### 2.2. Process Optimization for Lespedeza juncea Tea

#### 2.2.1. Tea Samples

Using traditional green tea processing techniques: Fresh leaves were picked, withered, pan-fired, and low-temperature-dried, resulting in rough tea.

Harvesting of fresh leaves: *Lespedeza juncea* was selected as the source material from the experimental field observation station of the Grassland Research Institute of the Chinese Academy of Agricultural Sciences in Dalad Banner, Ordos City, Inner Mongolia Autonomous Region. The fresh leaves were hand-picked in the budding stage in July 2025. After collection, the leaves were placed in well-ventilated bamboo containers. Leaves damaged by insects or disease were removed; only intact, fresh leaves were used as the raw material for the subsequent tea processing steps.

Withering: The fresh leaves were evenly spread on a 3–5 cm thick bamboo flat sieve and left to air dry for 5–6 h in a ventilated and dry room with an indoor temperature of 25–27 °C and relative humidity (RH %) of 27%. During this process, the leaves were turned twice to prevent uneven moisture content due to differences in water loss between the upper and lower layers.

Pan-firing: Using a traditional tea-firing wok, the withered leaves of *Lespedeza juncea* were spread out according to the leaf loading, roasting temperature, and roasting time. During the pan-frying process, the leaves were manually turned to ensure even heating and prevent localized overheating, which could cause the samples to scorch.

Rolling: After frying, the tea samples were placed on a flat bamboo tray. Once cooled to room temperature, the tea leaves were rolled by hand until a slight seepage of juices was observed.

Low-temperature drying: The rolled tea leaves were spread evenly on dry absorbent paper (thickness < 2 cm) and then placed in an oven for low-temperature drying (80 °C, 3 h) until the moisture content fell below 7%.

After drying, a portion of the samples was pulverized into powder and screened using a 60-mesh sieve for chemical composition analysis, while the unground portion served as samples for sensory evaluation. All prepared samples were stored at −80 °C.

#### 2.2.2. Single-Factor Trial Design

This experiment involved three factors: leaf loading (W), roasting temperature (T), and roasting time (t). Each factor was tested using six treatments, with three replicates per treatment. With consistent parameters and equipment across all other processing steps, the resulting *Lespedeza juncea* tea was thoroughly blended. The air-dried *Lespedeza juncea* was used as the control group sample (CK). A portion of the tea leaves was pulverized into powder to serve as samples for chemical composition analysis.

#### 2.2.3. Orthogonal Test Design for *Lespedeza juncea* Tea

Based on the single-factor experiments, an L9 (3^4^) orthogonal design was conducted with three factors, leaf loading (W), roasting temperature (T), and roasting time (t), each at three levels with three replications. Flavonoid content served as the evaluation metric to analyze primary and secondary factors influencing flavonoid levels, thereby determining optimal roasting conditions for the superior quality of *Lespedeza juncea* tea.

### 2.3. Sensory Evaluation

An evaluation panel comprising 12 professionals with experience in the sensory evaluation of tea engaged in quality assessment and conducted sensory evaluations of the *Lespedeza juncea* tea’s appearance, liquor color, aroma, taste, and leaf base following the Chinese National Standard (GB/T 23776-2018). First, 200 g of each tea sample was placed on a white square plate to assess the appearance of the dry leaves. Each tea sample (3.0 g) was infused with 150 mL of boiling water in a covered cup and steeped for 4 min. The infusion was filtered into a bowl and successively evaluated for liquor color, aroma, taste, and the characteristics of the infused leaves. A 100-point scale was used to calculate the scoring result, with appearance accounting for 25%, liquor color 10%, aroma 25%, taste 30%, and infused leaves 10% (Table S1).

### 2.4. E-Tongue Taste Analysis

The E-tongue system (402B Plus-EX, Insent Company, Atsugi, Japan) was used to evaluate the taste profiles of the tea samples. The sensory array included two reference electrodes and five chemical sensors, including a CA0 sensor for sourness, a C00 sensor for bitterness and aftertaste-B, an AAE sensor for umami, an AE1 sensor for astringency and aftertaste-A, and a CT0 sensor for saltiness. To ensure the stability and accuracy of test data, the E-tongue was calibrated in accordance with the calibration protocol (Table S2) provided by the equipment manufacturer. The tea samples were brewed according to Section 2.3. The tea liquors were cooled down to room temperature prior to analysis, as instructed by the instrument. Each tea sample was brewed twice, with each infusion measured three times. The average of these six measurements was used as the final E-tongue response.

### 2.5. Analysis of Volatile Compounds

Volatile compounds in *Lespedeza juncea* tea were analyzed using a GC-2030 model (Shimadzu, JAP) equipped with an MS detector and olfactory detection port (ODE-2030), as well as pre-processing using headspace solid-phase microextraction. 3-methyl-2-heptanone was used as the internal standard. This method was adapted from Feng W et al. with minor modifications [13]. The tea powder sample (1.0 g) was infused in 50 mL of boiling deionized water and bathed in boiling water for 4 min to obtain a tea liquor sample. Subsequently, 7.5 mL of the cooled liquor was transferred to a 15 mL extraction vial, and 2.25 g of sodium chloride was added, along with 5 µL of an internal standard solution (382 µg/mL). The sample was then adsorbed for 40 min at 40 °C using an extraction column (DVB/Carbon WR/PDMS). The sample was eluted for 3 min at an inlet temperature of 250 °C. Chromatography conditions: 40 °C (held for 3 min), 40–150 °C (at a rate of 8 °C/min), 150–260 °C (at a rate of 10 °C/min, held at 260 °C for 15 min). Mass spectrometry conditions: an ion source temperature of 220 °C, mass range of 35–350 *m*/*z*, and ionization energy of 70 eV. Volatile peaks were identified by matching the National Institute of Standards and Technology (NIST) mass spectrometry database and the retention indices (RI), determined by n-alkanes C7–C40). The odor activity value (OAV) is a commonly used indicator for measuring the contribution of a component to the overall odor. Calculations were performed based on the methods reported by Liu, Shen and Feng W et al. [14,15,16], with minor modifications.

(1) $$\mathrm{Relative}\;\mathrm{content}\;\mathrm{of}\;\mathrm{volatile}\;\mathrm{compounds}\;(µg/g)=\frac{\rho 1\times V1\times A2}{A1}/m$$

ρ1: Internal standard concentration (µg/L);

V1: Internal standard added volume (µL);

A2: Volatile compound peak area;

A1: Internal standard peak area.

(2) $$\mathrm{Relative}\;\mathrm{concentration}\;\mathrm{of}\;\mathrm{volatile}\;\mathrm{compounds}\;(µg/L)=\frac{\rho 1\times A2}{A1}$$

ρ1: Internal standard concentration (µg/L);

A2: Peak area of volatile compounds;

A1: Peak area of internal standard.

(3) Odor activity value (OAV)=COT

C: Relative concentration of volatile compounds (µg/L);

OT: Odor threshold (µg/L).

### 2.6. Analysis of Total Flavonoids, Tea Polyphenols, Soluble Sugars, Free Amino Acids, and Aqueous Extract

The total flavonoid content was determined using the NaNO_2_-Al (NO_3_)_3_ colorimetric method [17], with slight modifications. A total of 0.2 g of tea powder (accurate to 0.0001 g) was added to 5 mL of a 70% ethanol solution, and it was sonicated for 30 min. The samples were centrifuged at 3500 rpm for 10 min, and the supernatant was then transferred to a 10 mL volumetric flask. The above process was repeated, and the resulting supernatants were collected and diluted to the mark with 70% ethanol. A total of 1 mL of the test solution was transferred into a 10 mL volumetric flask, 4 mL of 70% methanol solution was added and shaken well, and 0.3 mL of NaNO_2_ was then added (5%). The solution was left to stand for 6 min. Subsequently, 0.3 mL of Al (NO_3_)_3_ (10%) was added, and the solution was again left to stand for 6 min. Following this, 4 mL of NaOH (4%) was added and diluted to the mark with deionized water. The samples were centrifuged at 3500 rpm for 10 min and then measured at 510 nm using rutin as the standard. Quantitative calculations were performed using the following formula:

(4) $$\mathrm{The}\;\mathrm{content}\;\mathrm{of}\;\mathrm{total}\;\mathrm{flavonoids}\;(\%)\;=\;\frac{(A1-A0)\times V\times 100}{m\times 1000\times 100}$$

A1: The measured concentration of the sample (mg/mL).

A0: The blank measurement concentration of the sample (mg/mL).

V: The extraction volume (mL).

m: The mass of the sample (g).

The tea polyphenol content was determined using the Folin–Ciocalteu method [18], with slight modifications. A total of 0.2 g of tea powder (accurate to 0.0001 g) was weighed in a 10 mL volumetric flask, and 5 mL of a 70% methanol solution was added and then extracted in a water bath at 70 °C for 30 min. The flask was shaken intermittently to ensure thorough extraction. The sample was then cooled down to room temperature and centrifuged at 3500 rpm for 10 min, and the supernatant was transferred to a 10 mL volumetric flask. This process was repeated, combining all the resulting solutions and diluting them to the mark. A total of 3 mL of the test solution was taken and diluted to 10 mL with a 70% methanol solution. Subsequently, 1 mL of this diluted solution was moved into a 10 mL volumetric flask, and 5 mL of phlorizin solution was added (10%). The solution was then left in the dark for 5 min. Following this, 4 mL of sodium carbonate solution (7.5%) was added, shaken well, and diluted to the mark with deionized water, and the solution was then left in the dark for 1 h. The samples were examined at 765 nm using gallic acid as the standard. Quantitative calculations were performed with the following formula:

(5) $$\mathrm{The}\;\mathrm{content}\;\mathrm{of}\;\mathrm{tea}\;\mathrm{polyphenols}\;(\%)\;=\;\frac{(A1-A0)\times V\times D\times 100}{m\times 1000\times 100}$$

A1: The measured concentration of the sample (mg/mL).

A0: The blank measurement concentration of the sample (mg/mL).

V: The extraction volume (mL).

D: The dilution factor of the sample.

m: The mass of the sample (g).

The soluble sugar content was determined using the anthrone–sulfuric acid method [19]. A total of 0.5 g of tea powder was weighed (accurate to 0.0001 g) in a 50 mL centrifuge tube. Subsequently, 10 mL of deionized water was added, and the mixture was extracted at 80 °C in a water bath for 30 min. The tube was then centrifuged at 3500 rpm for 10 min. The supernatant was transferred to a 10 mL volumetric flask, and the above procedure was repeated twice. The collected supernatants were used as the sample extracts. To precipitate the interfering substances, zinc acetate and potassium ferricyanide solutions were added to the extract sequentially. The solution was diluted to the mark, after which it was centrifuged at 4000 rpm for 5 min. A total of 3 mL of the supernatant was transferred to a 10 mL centrifuge tube and diluted to the mark with deionized water. Subsequently, 2 mL of this dilution was transferred to a 25 mL colorimetric tube. Following this, 12 mL of anthrone–sulfuric acid solution (0.2 mg/mL) was added and placed in a boiling water bath for 12 min. Finally, the solution was immediately cooled down to room temperature in an ice bath. Glucose was used as the standard and determined at 625 nm. Quantitative calculations were performed using the following formula:

(6) $$\mathrm{The}\;\mathrm{content}\;\mathrm{of}\;\mathrm{soluble}\;\mathrm{sugar}\;(\%)\;=\;\frac{(A1-A0)\times V\times D\times 100}{m\times 1000\times 100}$$

A1: The measured concentration of the sample (mg/mL).

A0: The blank measurement concentration of the sample (mg/mL).

V: The extraction volume (mL).

D: The dilution factor of the sample.

m: The mass of the sample (g).

The free amino acid and aqueous extract contents were determined according to the Chinese national standards (GB/T 8314-2013 and GB/T 8305-2013, respectively).

### 2.7. Metabolomic Analysis

Sample extraction: The tea powdered sample (0.100 g) was extracted with 500 µL of 80% methanol solution in an EP tube. The sample was vortexed; placed in an ice bath for 5 min; and then centrifuged at 15,000× *g*, 4 °C for 20 min. The supernatant was transferred to a methanol concentration of 53% by diluting it with mass spectrometry-grade water and centrifuged at 15,000× *g* and 4 °C for 20 min. The supernatant was then filtered through a 0.22 µm membrane before injection. Chromatographic conditions: Hypersil Gold column (C18); column temperature—40 °C; flow rate—0.2 mL/min; mobile phase A—0.1% formic acid; mobile phase B—methanol. Elution program: 0 min, 98% A, 2% B; 0–1.5 min, 98% A, 2% B; 1.5–3.0 min, 98% A, 2% B; 3–10 min, 15% A, 85% B; 10–10.1 min, 0% A, 100% B; 10.1–11 min, 98% A, 2% B; 11–12 min, 98% A, 2% B. Mass spectrometry conditions: Scan range selected at 100–1500 *m*/*z*. ESI source settings: spray voltage—3.5 kV; sheath gas flow rate—35 psi; tailgas flow rate—10 L/min; ion transfer tube temperature—320 °C; ion source RF level—60; tailgas heater temperature—350 °C; polarity—negative; MS/MS secondary scanning—data-dependent scanning.

### 2.8. The Analysis of Radical Scavenging Activity

DPPH radical scavenging assays were performed using the methods of Kebede et al. [20,21], with minor modifications. A 0.1 mmol/L DPPH radical solution was prepared using anhydrous ethanol as the solvent and DPPH powder as the solute. A stock solution (20 mg/mL) of tea liquor was prepared using a ratio of 1:50. This stock solution was diluted stepwise to obtain tea liquor samples of varying concentrations. Sample group: 100 µL of tea liquor + 100 µL of DPPH solution; control group: 100 µL of tea liquor and 100 µL of deionized water; blank group: 100 µL of deionized water and 100 µL of DPPH solution. Each tea solution concentration was replicated three times. The samples from each group were then thoroughly mixed and dispensed into a 96-well plate. Absorbance was measured at 517 nm using a microplate reader. The DPPH radical scavenging rate of the tea solution was then calculated using the following formula:

(7) $$\mathrm{DPPH}\;\mathrm{radical}\;\mathrm{scavenging}\;\mathrm{rate}\;(\%)\;=\;\frac{1-(A2-A1)}{A0}\times 100$$

A0: Absorbance value of blank group.

A1: Absorbance value of control group.

A2: Absorbance value of samples.

ABTS radical scavenging assays were performed using the methods of Sun et al. [21,22] with minor modifications. A K_2_S_2_O_8_ solution with a concentration of 140 mmol/L and an ABTS solution with a concentration of 7 mmol/L were prepared using deionized water. Subsequently, 178 µL of the K_2_S_2_O_8_ solution was mixed with 10 mL of the ABTS solution and stored in the dark for 16 h to generate ABTS radicals. The ABTS radical solution was adjusted with anhydrous ethanol to achieve an absorbance of 0.7 at 745 nm. The method for preparing tea liquor samples and the subsequent measurement procedures was analogous to that for the DPPH radical scavenging assay, with absorbance determined at 734 nm.

(8) $$\mathrm{ABTS}\;\mathrm{radical}\;\mathrm{scavenging}\;\mathrm{rate}\;(\%)=\frac{1-(A2-A1)}{A0}\times 100$$

A0: Absorbance value of blank group.

A1: Absorbance value of control group.

A2: Absorbance value of samples.

### 2.9. Data Analysis

The experimental data were organized and statistically analyzed using Excel. An orthogonal experimental design, a one-way analysis of variance (ANOVA), and multiple comparisons were then performed using SPSS 2024. The software and databases employed for metabolomics and volatile compound data processing include XCMS software (version 4.0), mzCloud (http://www.mzcloud.org/), mzVault, and the Masslist database, alongside the Linux (CentOS version 6.6) operating system and the NIST database (version 20), as well as R (version 3.4.3), Python (versio 3.5.0), and Origin software (Pro 2024). The analytical methods employed include PCA, cluster analysis, Pearson correlation analysis, and Partial Least Squares Discrimination Analysis (PLS-DA).

## 3. Results and Discussion

### 3.1. Process Optimization of Lespedeza juncea Tea

#### 3.1.1. Single-Factor Trial

**Figure 1:**
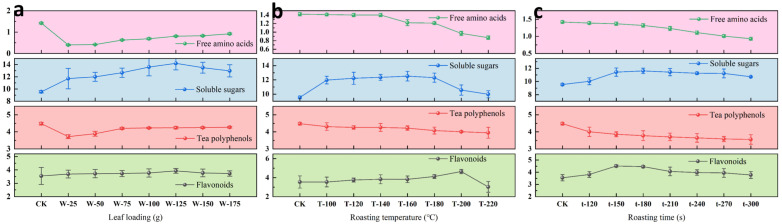
Major chemical components in *Lespedeza juncea* tea from single-factor trials. (**a**–**c**) represent the percentage content of flavonoids, tea polyphenols, soluble sugars, and free amino acids in *Lespedeza juncea* tea under different leaf loadings, roasting temperatures, and roasting times, respectively. Fixed factors: leaf loading = 100 g; roasting temperature = 160 °C; roasting time = 180 s.

As shown in Figure 1 and Tables S3a–S5a and S3b–S5b, the contents of flavonoids and soluble sugars initially increased and then decreased with an increase in roasting temperature, the extension of roasting time, and an increase in leaf loading. Tea polyphenol and free amino acid contents decreased as the roasting temperature and time increased. As leaf loading increased, free amino acids showed an upward trend, while tea polyphenols exhibited an initial rise followed by a decline. The flavonoid and soluble sugar contents reached their highest levels when the leaf loading (W) level was 125 g, at 3.93% and 14.20%, respectively. The highest flavonoid and soluble sugar contents were achieved at a roasting temperature (T) of 160 °C, at (4.65 ± 0.22)% and (12.52 ± 0.68)%, respectively. Flavonoid content peaked at (4.51 ± 0.10)% at a roasting time (t) of 150 s, while soluble sugar content reached the maximum of (11.62 ± 0.40)% at 180 s.

Flavonoids and tea polyphenols are key flavor compounds that contribute to the astringency and bitterness of tea liquor [23], giving it a complex taste profile. Flavonoids have excellent antioxidant properties and can have anti-inflammatory and anti-aging effects on the body [24,25]. Free amino acids and soluble sugars are crucial determinants of tea’s palatability. Depending on their type, free amino acids impart umami, bitterness, or sweetness to the tea liquor, while soluble sugars reduce astringency and bitterness [26,27]. The ratio of tea polyphenols to free amino acids (PAR) affects the sweetness of the tea, with a lower ratio producing a more pronounced sweetness and playing a crucial role in enhancing the tea’s taste [28]. Thus, the contents of flavonoids, tea polyphenols, free amino acids, and soluble sugars are key factors that influence the flavor of tea liquor. Amino acids and reducing sugars are crucial precursors for the Maillard reaction, generating volatile aroma compounds [13,29]. Thus, reducing the content of free amino acids and soluble sugars during tea processing facilitates the synthesis of these flavor compounds.

#### 3.1.2. Orthogonal Test

An orthogonal design was employed to investigate the effects of three factors [30,31,32], leaf loading (W = 125 g, 150 g, and 175 g), roasting temperature (T = 160 °C, 180 °C, and 200 °C), and roasting time (t = 150 s, 180 s, and 210 s), on the basis of single-factor experiments. The parameters of the preliminary orthogonal experiments are shown in Table S6. Based on prior research indicating that the primary active constituents in *Lespedeza juncea* are flavonoid compounds, which possess excellent antioxidant properties, and considering that flavonoids are typically associated with potent antioxidant and anti-inflammatory effects, it can be hypothesized that these compounds may improve oxidative status, mitigate elevated reactive oxygen species levels, and reduce oxidative stress [4,33,34]. Furthermore, they may influence inflammatory mediators and pathways, thereby exerting effects on metabolic disorders [35]. Thus, flavonoid content was used as the evaluation metric to identify the factors that significantly influenced the outcomes of the experiments. This approach determined the main factors affecting the quality of *Lespedeza juncea* tea and identified the optimal processing conditions.

The range analysis results in Table S6 revealed that the primary factors affecting the total flavonoid content of *Lespedeza juncea* tea were roasting temperature (T), roasting time (t), and leaf loading (W), in the order of influence. Previous studies indicated that pan-firing temperature plays a crucial role in shaping and regulating the green tea flavor profile. High-temperature fixation rapidly inactivates polyphenol oxidase within the leaves, thereby preserving a greater proportion of their chemical constituents. Additionally, an appropriate pan-firing duration facilitates thermal degradation and Maillard reactions, thus resulting in the increased production of volatile and non-volatile compounds [29,36,37,38]. Leaf loading is a factor that must be considered for specific tea pan-frying tools. Excessive leaf loading may result in the uneven heating of the leaves, thus leading to insufficient chemical conversion. If the leaf loading rate is too low, it may result in rapid water loss and an insufficient thermochemical reaction. An appropriate leaf loading rate can generate steam in the pot, thereby promoting the accumulation of soluble sugars and the conversion of amino acids and tea polyphenols. The optimal roasting conditions for *Lespedeza juncea* tea consisted of W_2_T_3_t_2_, a leaf loading level of 150 g, a roasting temperature of 200 °C, and a roasting time of 180 s. To facilitate the subsequent data analysis, the orthogonal experimental groups are denoted by the following letters: A (160 °C, 150 s, 125 g), B (160 °C, 180 s, 175 g), C (160 °C, 210 s, 150 g), D (180 °C, 150 s, 150 g), E (180 °C, 180 s, 125 g), G (180 °C, 210 s, 175 g), H (200 °C, 150 s, 175 g), I (200 °C, 180 s, 150 g), and K (200 °C, 210 s, 125 g).

### 3.2. Results of Sensory Evaluation and Taste Assessment

**Figure 2:**
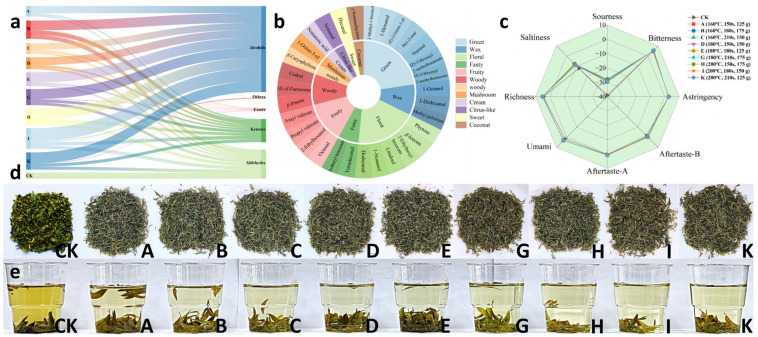
Sensory evaluation and flavor profile of *Lespedeza juncea* tea. (**a**) Major categories of volatile compounds detected in each group. (**b**) Odor classification chart for volatile compounds. (**c**) Results of electronic tongue analysis of taste profile in each group. (**d**,**e**) Appearance and liquor in each group.

The sensory evaluation results are shown in Table 1. Groups I, K, and H achieved the highest overall scores of 90.9, 90.2, and 89.2, respectively, while group CK scored the lowest with 79.5 points. In terms of appearance, we can see from Figure 2d that group CK exhibited green coloration, while the remaining groups displayed varying degrees of brown hues. Of these, the G, I, and K groups were the darkest brown, followed by the D, E, and H groups, with the A, B, and C groups being the lightest. Previous studies have shown that the visual color and brightness of brewed tea leaves are directly influenced by the content of chlorophyll and carotenoids [39,40]. As this study did not analyze these indicators, further investigation is warranted. Secondly, in terms of aroma and flavor, groups H, I, and K exhibited a subtle roasted fragrance and earned higher scores, and group I achieved the highest score, while groups D, E, and G had a slightly milder aroma and scored the second highest. Group CK, however, displayed a distinct grassy aroma and received the lowest score. Regarding liquor color, the CK group had a deep, slightly cloudy liquor, and group A was also slightly cloudy. The remaining groups displayed pale yellow or pale green liquors that were all clear and translucent (Figure 2e). In terms of flavor profile, groups H, I, and K exhibited slight bitterness and astringency with a hint of sweetness, delivering a mellow and rich taste. The color and flavor intensity of tea liquor correlate with the presence and concentration of chemical constituents in the tea leaves. For example, flavonoids affect the brightness of the liquor’s color and can make the brew slightly bitter [41,42]. The CK group tasted unripe, with pronounced grassy notes and an inferior texture. The remaining groups offered a lighter flavor accompanied by subtle grassy aromas. Relevant research indicates that pan-firing can effectively reduce the grassy odor in tea leaves due to its unique method of heat conduction [43]. Finally, post-infusion leaf base scores ranged from 77 to 80 across all groups, showing little variation. After steeping, the CK group’s leaf base appeared to have a tender green color, while the others exhibited a tender yellowish-green hue.

According to the E-tongue measurement results (Figure 2c), the umami value of the tea infusion in group CK was lower, which was inconsistent with its relatively high free amino acid content. This discrepancy may be attributed to differences in the proportion of various amino acids and the distinct taste thresholds of different flavor-enhancing amino acids. Studies indicate that amino acids such as aspartic and glutamic acids impart an umami flavor, while serine, alanine, and proline contribute sweetness. Conversely, arginine, histidine, and leucine produce bitterness [26,44]. However, this study did not measure the specific types of amino acids present in *Lespedeza juncea* tea. Further research is needed to explore the relationship between the types and concentrations of amino acids in tea and its taste. Group CK exhibited higher astringency values in the tea liquor, which may be due to its higher polyphenol content. Relevant research indicates that polyphenols contribute to the astringency of tea liquor, and polyphenolic compounds are present in higher concentrations in fresh tea leaves [45]. Groups H, I, and K exhibited slightly more bitterness than other groups, enriching the tea’s flavor profile. This may be attributed to the relatively higher flavonoid content in these groups. Studies indicate that due to their low threshold range (1.15 × 10^−3^ ~ 19.8 µmol/L), flavonoids and flavonoid glycosides are highly detectable flavor compounds that impart bitterness to tea liquor [38,46].

Based on sensory evaluation and the E-tongue measurement results, the groups with the highest scores were I, K, and H. Collectively, these findings indicate that the sensory qualities and flavor of the *Lespedeza juncea* tea produced under the conditions of H, I, and K were slightly superior to those of the other groups.

### 3.3. Results of Volatile Compound Determination

To investigate flavor differences in *Lespedeza juncea* tea produced under varying roasting conditions, headspace solid-phase microextraction (HS-SPME) was used to identify volatile compounds in the tea liquor via gas chromatography–mass spectrometry (GC-MS). The qualitative determination of volatile compounds employs n-alkanes, and qualification was achieved through mass spectrometry (MS) and the retention index (RI). Thirty-one volatile compounds with a similarity index (SI) above 80% and RI ± 50 were identified by matching the NIST database. These compounds were primarily alcohols, aldehydes, ketones, and esters (Figure 2a). Among these, there were ten alcohol compounds, including 1-octen-3-ol with a strong mushroom aroma, 1-octanol and linalool with floral and fruity notes, and 1-dodecanol with a buttery fragrance; eight aldehyde compounds, including fatty-scented octanal, orange-scented and rose-scented nonanal, and sweet-scented and floral-scented decanal; and seven ester compounds, including amyl valerate with a ripe apple aroma and isobutyl benzoate with a buttery and fruity fragrance. The two ketone compounds were *β*-ionone and phytone, which have floral and fruity aromas. The remaining four compounds included alkenes, phenols, and acids, consisting of *β*-pinene, (*E*)-*β*-farnesene, caryophyllene, and nonanoic acid. These primarily contribute herbal aromas along with notes of hay and wood (Figure 2b).

To further analyze the differences in volatile compounds between groups, Table S7 summarizes the volatile compounds detected in each group. Nine compounds were detected as common across all groups, comprising 1-octen-3-ol, n-octanol, 1-nonanol, dodecyl alcohol, n-octanal, nonanal, decanal, *β*-ionone, and pentyl valerate. These compounds contribute varying degrees of floral, green, fruity, and oily aromas to the tea, and they may be the main aromatic compounds responsible for the characteristic aroma of *Lespedeza juncea* tea. Shu et al. [47] classified green tea into three types based on their distinct aroma characteristics: the chestnut aroma, floral aroma, and fresh aroma types. The volatile compounds found in *Lespedeza juncea* tea include 1-octen-3-ol, nonanal, decanal, *β*-ionone, and linalool. Linalool is a group I-specific volatile compound and a primary aroma-forming substance in chestnut-scented green tea [48]. During the detection of volatile substances in combination with a sniffing test, a pronounced mushroom aroma was detected approximately ten minutes into the experiment. This aligns with the scent of the compound 1-octen-3-ol, which was also detected around this time. After approximately 20 min, a strong floral–fruity aroma emerged, accompanied by a subtle sweet fragrance. These odors closely resembled those of *β*-ionone and dodecyl alcohol, which were also detected at this time.

2-methyl-1-butanol, linalool, *β*-pinene, and nonanoic acid were detected only in group I, contributing grassy and cocoa notes, floral–fruity aromas, and woody scents. Phytone, which imparts a subtle floral–fruity aroma, was detected only in group C. (*E*)-*β*-farnesene and caryophyllene were detected only in groups K and H, respectively, contributing nutty and woody aromas.

To investigate the aroma differences among the tea samples, a relative quantitative analysis was conducted using internal standards for the detected volatile compounds. The calculations revealed that group I has the highest content of 1-octen-3-ol, followed by groups K and H, and n-octanol, followed by groups H and E, as well as the highest concentration of 1-nonanol, followed by groups B and K. Group K has the highest concentration of 1-dodecanol, followed by groups I and C. Meanwhile, group I has the highest content of n-octanal, followed by groups G and K. Group CK has the highest concentration of nonanal, followed by groups B and H, and the highest content of decanal, followed by groups K and I. Group I also has the highest content of *β*-ionone, followed by groups H and K. Finally, group A has the highest content of amyl valerate, followed by groups D and E. According to previous studies, the odor activity value (OAV) is a characteristic indicator used to evaluate the distinctive aroma of tea liquor [49]. The OAVs of volatile compounds in *Lespedeza juncea* tea were determined based on their relative content and the odor thresholds (OTs). The OTs cited in this document were derived from previously published research articles on tea flavor. Subsequently, the OAV of the volatile compounds in *Lespedeza juncea* tea was calculated. As shown in Tables S8 and S9 [44,49,50], the OAV of 1-nonanol ranges between 0.1 and 1 among the volatile compounds in *Lespedeza juncea* tea, thus playing a supplementary role in terms of aroma. The OAV of 1-dodecanol < 0.1, which indicates that it contributes minimally to the tea’s aroma. The OAV of all other compounds exceeds 1, thus indicating that these compounds significantly contribute to the aroma of the tea and are key to its overall fragrance. Group I exhibited a higher OAV for compounds such as 1-octen-3-ol and *β*-ionone, which impart mushroom and floral notes, respectively. *β*-ionone had a relatively high OAV in group I. Moreover, the OAV of linalool and nonanoic acid exceeded 1, and both are unique to group I. This indicates that these two compounds are key contributors to the tea aroma of group I. This finding aligns with Zhang et al.’s determination of volatile metabolites in green tea processed by pan-firing across different seasons, which identified 1-octen-3-ol and *β*-ionone as primary components shaping the tea’s aroma [51]. Additionally, Table S10 [44,52,53] summarizes the volatile compounds unique to each group. Linalool and nonanoic acid exhibit an OAV > 1 in group I, imparting floral, fruity, and buttery aromas. This indicates that linalool and nonanoic acid are the characteristic aroma compounds that are specific to group I and contribute to its distinctive fragrance. Chen et al. investigated the aroma components in Guzhang Maojian tea at different withering temperatures using HS-SPME-GC-MS and found that decanal, nonanal, and linalool are key components that contribute to its characteristic aroma [54]. Additionally, Zhang et al. analyzed the aroma compounds of Xinyang Maojian tea of different grades and found that *β*-ionone exhibited a positive correlation with tea grade [55]. It is thus suggested that *β*-ionone is an important aroma compound for grading tea. Therefore, the volatile compounds present in each group may constitute the primary components that give the *Lespedeza juncea* tea its characteristic aroma. Furthermore, the roasting conditions of group I are more conducive to the accumulation of these aromatic compounds, thereby making it a more suitable processing condition for *Lespedeza juncea* tea production.

Based on the above results, group I had the highest number of unique volatile compounds, and its total volatile compound content was relatively high (628.92 µg/g). Based on the results of the sniffing test, a comparison of the relative content and OAVs of 1-octen-3-ol and *β*-ionone, which are the two compounds primarily responsible for the aroma of the *Lespedeza juncea* tea liquor, revealed that the relative content (OAV) of 1-octen-3-ol in groups I, K, and H was 365.49 µg/g (73.04), 338.34 µg/g (71.44), and 336.93 µg/g (67.39), respectively, and the relative content (OAV) of *β*-ionone was 8.38 µg/g (239.54), 5.42 µg/g (162.43), and 7.85 µg/g (223.99), respectively. This indicates that under the experimental conditions, the three groups of *Lespedeza juncea* tea had similar concentrations of the main aroma components. Groups H, I, and K shared the same roasting temperature of 200 °C. Therefore, it is inferred that a roasting temperature of 200 °C may be conducive to the production of aroma compounds in *Lespedeza juncea* tea. Adjusting the roasting time and leaf loading appropriately can significantly enhance the generation of aroma compounds in *Lespedeza juncea* tea.

### 3.4. Results of Analysis of Major Chemical Components

**Figure 3:**
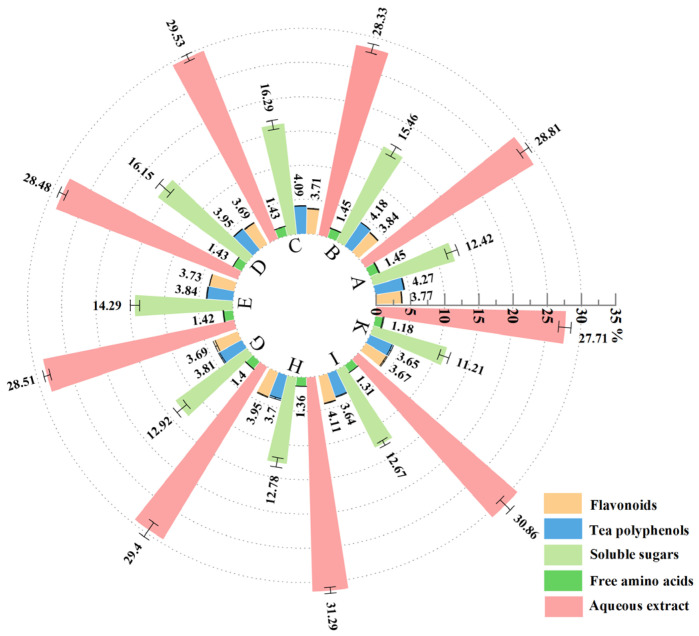
Chemical component content in *Lespedeza juncea* tea under different roasting conditions.

Table S11 and Figure 3 show the chemical composition of *Lespedeza juncea* tea under different roasting conditions. Analysis revealed that the free amino acid content in treatment groups I and K showed no significant difference from group H but was significantly lower than that in the other groups. Group A exhibited the highest tea polyphenol content at (4.27 ± 0.07)%, which showed no significant difference from group B but was significantly higher than that in other groups. The groups with the lowest PAR levels were E < G < H < D < I. The aqueous extract content ranged from (27.71 ± 0.08)% to (31.29 ± 0.45)%. Among the treatment groups, group H exhibited the highest aqueous extract content at (31.29 ± 0.45)%, showing no significant difference compared to group I but significantly higher than that in the other groups. Group C had the highest soluble sugar content at (16.29 ± 0.66)%, showing no significant difference from groups B and D but significantly higher than that in the other groups.

The analysis of the results from the single-factor and orthogonal experiments on *Lespedeza juncea* tea revealed that, under the experimental conditions, the primary factor influencing the flavonoid content of the tea was roasting temperature (T), followed by roasting time (t) and leaf loading (W). Groups H and I had the highest aqueous extract content, which is responsible for the richness and depth of the tea liquor [56]. Group I had the highest flavonoid content in *Lespedeza juncea* tea, followed by groups H and B in the orthogonal experiment. This aligns with the single-factor experiments showing a higher flavonoid content at roasting temperatures between 160 °C and 200 °C and is consistent with Lin et al.’s findings that under identical roasting temperatures and leaf loadings, appropriately extending the roasting time promotes flavonoid accumulation in loquat flower tea [57]. Li et al. investigated the physicochemical properties and sensory flavor of barley tea by optimizing the roasting process, finding that appropriately increasing roasting temperature and duration promotes the accumulation of flavonoid compounds [58]. The content of tea polyphenols and free amino acids decreased as the roasting temperature and duration increased, while the content of soluble sugars initially increased before decreasing. These results are consistent with previous research on the changes in polyphenolic compounds in tea leaves [59,60]. Qiu et al. investigated major changes in the chemical composition of tea during processing and observed a decreasing trend in tea polyphenols and free amino acids, while an initial increase followed by a decrease was observed for the content of soluble sugars, along with particularly significant changes during heat processing [61].

### 3.5. Results of Metabolomics

**Figure 4:**
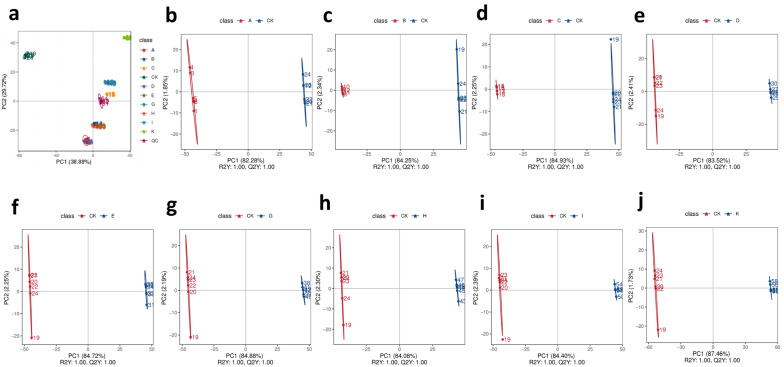
PCA diagram (**a**), PLS-DA diagram for each treatment group relative to CK group (**b**–**j**), R^2^Y and Q^2^Y values for all comparison pairs exceeded 0.5, indicating robust model establishment.

**Figure 5:**
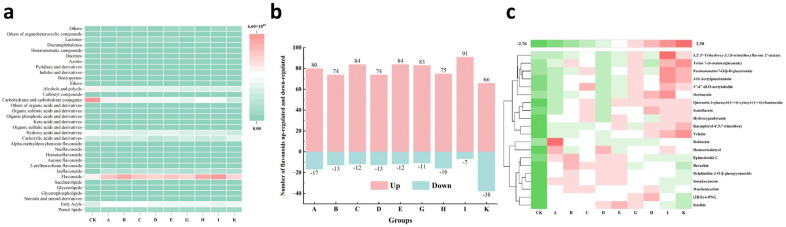
Changes in flavonoid metabolites in *Lespedeza juncea* tea under different roasting conditions. (**a**) Changes in different types of metabolites. (**b**) Number of up- and down-regulated flavonoid metabolites in each group. (**c**) Changes in differentially expressed metabolites common to all groups compared to group CK.

A total of 1788 metabolic features were detected in negative ion mode through database comparisons using mzCloud, mzVault, and Masslist. In the Section 2.7, four quality control (QC) samples were introduced. These samples were prepared by pooling the test samples. Correlation analysis was performed on the QC samples, revealing correlations between the four samples ranging from 0.993 to 0.996 (Figure S1). This indicates good correlation, demonstrating that the measurement data are stable and reliable. All metabolites putatively annotated based on MS1 and MS2 database matching, MS2, and provided database matching scores (>0.5). During data processing, the mass spectrometry error was ≤5 ppm, ultimately confirming the metabolites. Through principal component analysis, the principal components (PC1 and PC2) accounted for 68.6% of the total variance, with the first principal component projection value at 38.88% (Figure 4a). The samples clustered into distinct groups, with the CK and K groups positioned farther apart from the other groups, indicating greater metabolic differences. Overall, the pattern shows clustering within groups and relative dispersion between individual groups. Ignoring leaf input quantity, the three groups with identical roasting temperatures are ABC, DEG, and HIK. As time increased, the spatial distance between groups became relatively more dispersed, thus indicating that roasting time significantly influences their metabolites. This corresponds to the result R = 0.2 for roasting time in the orthogonal experiment. Meanwhile, the three groups with identical roasting times are ADH, BEI, and CGK. As temperature increased, spatial proximity was observed between groups A and D, groups B and E, and groups C and G, even overlapping in some cases. Conversely, group H was relatively distant from AD, group I from BE, and group K from CG. This indicates that a roasting temperature of 200 °C exerted a greater influence on sample metabolites than 160 °C or 180 °C, which is consistent with the results of the orthogonal experiment, in which roasting temperature R > 0.2. Conversely, closer distances suggest similarities in metabolic profiles among the groups. Sample category prediction was achieved through supervised statistical analysis—Partial Least Squares Discrimination Analysis (PLS-DA). As shown in Figure 4b–j, PLS-DA models were established for each group versus the CK group, and the validation results for these models were ranked based on 200 shuffled modeling runs. The R^2^Y and Q^2^Y values for all comparison pairs exceeded 0.5, thus indicating robust model establishment. Furthermore, permutation tests were used to evaluate model robustness and avoid overfitting. The results indicated that the model is not overfitted and can adequately describe the samples (Figure S2). Cluster analysis revealed that flavonoid metabolites were up-regulated compared to group CK (Figure 5a). A further analysis of changes in flavonoid metabolites across groups showed that each group exhibited a different number of up-regulated and down-regulated flavonoid metabolites compared to group CK (Figure 5b). Group I exhibited the most up-regulated metabolites and the fewest down-regulated metabolites, while group K exhibited the least up-regulation and the most down-regulation. These results indicate that the roasting conditions corresponding to group I may be more conducive to the accumulation of flavonoids, and this finding is consistent with the chemical composition analysis results showing the highest total flavonoid content in group I. A total of 334 flavonoids were detected across all groups. After screening (VIP > 1, P < 0.05, FC > 2, and FC < 0.5), 20 metabolites with significant differences were identified across groups. Heatmap analysis (Figure 5c) revealed that differentially expressed metabolites in all groups were generally up-regulated compared to group CK, with only a few being down-regulated. Groups E, I, and K exhibited relatively more flavonoid metabolites and higher degrees of up-regulation.

As shown in Table S12, the differential metabolites in group I consist of flavonoid glycosides, 3″,4″-di-*O*-acetylafzelin (CAS: 77307-50-7), formononetin-7-*O*-*β*-*D*-glucuronide (CAS: 18524-03-3), scutellarein (CAS: 27740-01-8), and tricin 7-(6-malonylglucoside) (PubChem CID: 122391240), and *O*-methylated flavonoids, velutin (CAS: 25739-41-7), homoeriodictyol (CAS: 446-71-9), hydroxygenkwanin (CAS: 20243-59-8), and 5,2′,5′-trihydroxy-3,7,8-trimethoxyflavone-2′-acetate (PubChem CID: 44259911). Two flavones were also identified, kaempferol-4′,5,7-trimethoxy (CAS: 1098-92-6) and herbacetin (CAS: 527-95-7), as well as one flavan, 3-*O*-acetylpinobanksin (CAS: 52117-69-8). The metabolites significantly up-regulated in group K were identical to those in group I, except for herbacetin and homoeriodictyol. Among these, 3″,4″-di-*O*-acetylafzelin is a compound capable of selectively inhibiting ribosomal S6 kinase (RSK) activity. Owing to its highly selective inhibitory effect on RSK, it is regarded as a potential natural compound for anticancer applications [62,63,64]. Scutellarin inhibits the proliferation and migration of ovarian cancer cells by targeting METTL5 [65]. Reducing AP-1 activation prevents obesity-induced renal fibrosis [66] and induces apoptosis via the ERK1/2 and AKT pathways, thereby improving non-alcoholic fatty liver disease [67]. As a natural flavonoid compound, velutin exhibits melanin-inhibiting, anti-inflammatory, and intervertebral disk degeneration-protective effects [68,69]. It demonstrates potent efficacy in suppressing the production of inflammatory mediators [70]. Hydroxygenkwanin (HGK) inhibits peritoneal metastasis in colorectal cancer by modulating macrophage polarization. Herbacetin exhibits broad-spectrum antiviral, anti-fibrotic, and anti-ferroptotic properties, thus suppressing viral enzyme activity, alleviating pulmonary fibrosis, and protecting neurons from ferroptotic damage [71]. 3-O-acetylpinobanksin may exert anti-osteoporotic effects by targeting MAPK1/3, RXRA, AKT1, ESR1, and STAT3 to modulate the AGE-RAGE pathway [72].

Compared to group CK, only groups A, E, and K exhibited unique differential metabolites, totaling 15 compounds primarily consisting of flavone glycosides, as shown in Table 2. Group K had the most unique differential metabolites (12), of which only one compound, 5,7,3′,5′-Tetrahydroxy-3,6,8,4′-tetramethoxyflavone (PubChem CID: 44260071), was up-regulated. The others were all down-regulated, including myricetin 3-(6″-*p*-coumaroylglucoside), vitexin 7-*O*-sulfate, quercetin 3-(6″-caffeylgalactoside), etc. Two unique differentially expressed metabolites were detected in group A, both of which were down-regulated, while one unique differentially expressed metabolite that was up-regulated was detected in group E: epigallocatechin 3-*O*-*p*-coumarate (CAS: 89013-65-0).

An analysis of non-targeted metabolomic data revealed that flavonoid compounds were up-regulated compared to the CK group and enriched in group I. This aligns with the determination of total flavonoid content in the chemical composition, which indicates that the flavonoid content of *Lespedeza juncea* leaves increases under appropriate roasting conditions. Moreover, most metabolites have been documented in the literature as being detected in plants, exhibiting anti-inflammatory and antiviral effects. Certain metabolites also represent potential therapeutic agents for Alzheimer’s disease and cancer (Table S12). A few metabolites lack corresponding literature reports, thus necessitating further investigation into their metabolic pathways and biological significance. Group K had the most down-regulated unique differential metabolites, predominantly consisting of flavonoid glycosides, including myricetin, quercetin, orientin, and vitexin. Among these, myricetin and quercetin are the primary flavonoid glycosides in tea leaves [75,76], while orientin and vitexin are the primary flavonoids in *Lespedeza juncea* [4]. The down-regulation of flavonoid glycosides may be associated with prolonged high-temperature pan-frying conditions. Studies have shown that high-temperature fixation causes the thermal degradation of flavonoid glycosides in tea leaves, thus resulting in decreased content [77,78,79].

### 3.6. Results of Antioxidant Activity Measurements

#### 3.6.1. The Scavenging Activity of DPPH Radicals

**Figure 6:**
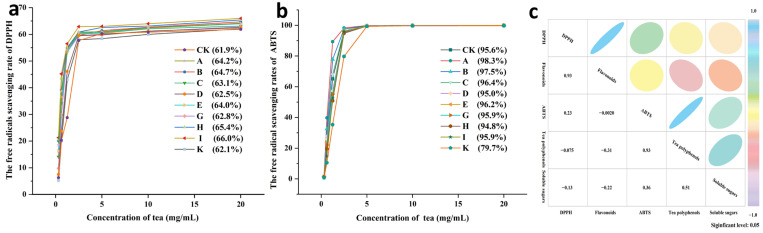
Radical scavenging rates of different concentrations of *Lespedeza juncea* tea. (**a**) Panel data represent DPPH radical scavenging rate at 20 mg/mL. (**b**) Panel data represent ABTS radical scavenging rate at 2.5 mg/mL. (**c**) Panel shows correlation between radical scavenging effects and chemical components in *Lespedeza juncea* tea.

Experiments measuring the scavenging activity of tea liquor against DPPH radicals at different concentrations revealed that the scavenging rate of each liquor increased with rising concentration, peaking at 20 mg/mL. As shown in Figure 6a, the scavenging rates of all groups were higher than those of group CK. Group I exhibited the highest scavenging rate at 66.0%, followed by groups H, B, and A with rates of 65.4%, 64.7%, and 64.2%, respectively.

#### 3.6.2. The Scavenging Activity of ABTS Radicals

As shown in Figure 6b, the rate at which each tea liquor scavenged ABTS radicals increased as the tea concentration rose. At a concentration of 5 mg/mL, all teas achieved a scavenging rate of over 99%. Figure 6b shows the radical scavenging rates of each tea group at a concentration of 2.5 mg/mL against ABTS radicals. Groups A, B, and C exhibited higher scavenging rates of 98.3%, 97.5%, and 96.4%, respectively, while groups D, H, and K exhibited lower scavenging rates. All other groups showed higher scavenging rates than the CK group. At a concentration of 1.25 mg/mL, the rates at which the tea liquor scavenged radicals differed significantly between groups. Groups A, B, and C exhibited higher rates than group CK, while all the other groups showed lower rates.

The data above indicates that the tea liquor of *Lespedeza juncea* possesses favorable antioxidant activity. The scavenging activity of each group against DPPH and ABTS radicals increased as the tea liquor concentration rose. Correlation analysis revealed that the scavenging rate of ABTS radicals by tea liquor showed a significant positive correlation (r = 0.93) with tea polyphenol content, while the scavenging rate of DPPH radicals showed a significant positive correlation (r = 0.93) with flavonoid content (Figure 6c). This aligns with Zhang et al.’s findings regarding the relationship between chemical constituents and antioxidant activity in Eucommia leaf tea [51]. Furthermore, relevant studies indicate that tea polyphenols and flavonoid compounds have strong antioxidant properties [78]. Our preliminary research also showed that *Lespedeza juncea* is rich in flavonoid compounds and that its extracts exhibit free radical scavenging and antioxidant properties [33,34,80,81,82,83,84,85,86,87,88,89,90,91]. These results suggest that proper roasting conditions can increase the antioxidant activity of *Lespedeza juncea* tea.

## 4. Conclusions

This study optimized the processing conditions for *Lespedeza juncea* tea using orthogonal experiments combined with sensory and metabolomic analyses. The results indicated that *Lespedeza juncea* tea produced with a leaf loading level of 150 g, roasting temperature of 200 °C, and roasting time of 180 s exhibited intact leaves with a deep green color. After brewing, the tea liquor displayed a clear and translucent appearance, with a refreshing taste and a distinctive, harmonious aroma. The tea sample exhibited high flavonoid content under these conditions, thus demonstrating excellent scavenging effects against DPPH and ABTS radicals and significant flavonoid metabolite enrichment. Volatile compounds are diverse and abundant, providing an excellent foundation of material for creating distinctive tea beverages. A total of 31 volatile aroma compounds were detected via HS-SPME-GC-O-MS and LC-MS. The air-dried leaves contained primarily aldehydes, while the pan-fired samples primarily featured alcohols, including 1-octen-3-ol, n-octanol, 1-nonanol, and dodecyl alcohol, accompanied by n-octanal, nonanal, decanal, *β*-ionone, and pentyl valerate. The overall aroma profile closely resembles that of chestnut-scented green tea, and it exhibits excellent flavor characteristics. This study systematically elucidated the quality formation patterns of *Lespedeza juncea* tea produced using green tea processing techniques, employing modern tea processing theories and analytical methods. Given the abundance of beneficial flavonoid compounds in *Lespedeza juncea* tea, future research may explore natural flavonoid extraction or health beverage applications to maximize its potential value. This work not only revives an ancient tea tradition but also provides a scientific basis for developing novel functional teas.

## Figures and Tables

**Table 1:** Sensory evaluation results for *Lespedeza juncea* tea.

Indicator	CK	A	B	C	D	E	G	H	I	K
Appearance (Shape, Tenderness, Color, Integrity, Purity)	90	91	91	90	90	90	90	90	92	91
Tea liquor (Color Type, Intensity, Brightness, Clarity)	80	92	92	93	92	90	90	89	90	91
Aroma (Type, Intensity, Purity, Persistence)	78	85	86	87	87	87	87	90	92	91
Taste (Strength, Body, Mellow/Astringent, Purity/Foreign Notes, Freshness/Dullness)	70	86	86	86	86	87	87	91	90	92
Leaf base (Tenderness, Color, Brightness/Darkness, Uniformity)	85	79	78	77	79	78	80	80	80	80
Weighted total score	79.5	86.9	87.05	87.05	87.15	87.15	87.35	89.2	90.9	90.2

**Table 2:** Types of unique flavonoid differentially expressed metabolites detected in each group.

Group	No.	Name	CAS No.	Class	Level	Score	Mass Error	FC	Up/ Down
A-CK	1	Mitoflaxone	87626-55-9	Flavones	1	0.56	1.07	0.01	down
	2	Baohuoside I	55395-07-8	Flavonoid glycosides	3	-	1.33	0.04	down
E-CK	3	Epigallocatechin 3-*O*-*p*-coumarate	89013-65-0	Flavans	2	0.57	1.23	2.25	up
K-CK	4	Myricetin 4′-methyl ether 3-*O*-*β*-*D*-galactopyranoside	PubChem CID 44259503	Flavonoid glycosides	3	-	0.82	0.33	down
	5	Vitexin-2″-*O*-rhamnoside	64820-99-1	Flavonoid glycosides	1	0.99	1.24	0.48	down
	6	Phlorizin	60-81-1	Flavonoid glycosides	1	0.73	0.42	0.46	down
	7	Orientin	28608-75-5	Flavonoid glycosides	1	0.97	0.92	0.35	down
	8	4′-*O*-methyllucenin II	98813-28-6	Flavonoid glycosides	3	-	1.32	0.30	down
	9	Quercetin 3-*O*-*β*-(6″-*O*-(*E*)-*p*-coumaroylglucoside)-7-*O*-*β*-glucoside	LM ID: LMPK12112163 [73]	Flavonoid glycosides	1	0.83	0.65	0.19	down
	10	Isorhamnetin-3-galactoside-6″-rhamnoside	MSBNK-RIKEN-PR303239 [74]	Flavonoid glycosides	1	0.56	1.07	0.46	down
	11	Myricetin 3-(6″-*p*-coumaroylglucoside)	PubChem CID: 44259467	Flavonoid glycosides	1	0.75	0.22	0.14	down
	12	Vitexin 7-*O*-sulfate	PubChem CID: 44257740	Flavonoid glycosides	1	0.56	1.07	0.24	down
	13	Quercetin 3-(6″-caffeylgalactoside)	PubChem CID: 44259123	Flavonoid glycosides	3	-	1.33	0.23	down
	14	Myricetin 3-glucuronide	PubChem CID: 5487413	Flavonoid glycosides	2	0.57	1.23	0.11	down
	15	5,7,3′,5′-Tetrahydroxy-3,6,8,4′-tetramethoxyflavone	PubChem CID: 44260071	O-methylated flavonoids	3	-	0.82	6.19	up

Level 1: The metabolite in the sample matches the database in terms of all three parameters (MS1, MS2, and RT). Level 2: The metabolite in the sample matches the database in terms of both MS1 and MS2. Level 3: The metabolite in the sample matches the database at MS1.

## Data Availability

The original contributions presented in this study are included in the article/Supplementary Materials. Further inquiries can be directed to the corresponding authors.

## Supplementary Materials

The following supporting information can be downloaded at: https://www.mdpi.com/article/10.3390/foods15061066/s1, Table S1: Sensory evaluation scoring criteria and basis for *Lespedeza juncea* tea; Table S2: Mass concentration of standard solution and reference solution (per 1 L).Table S3a: Chemical component content in *Lespedeza juncea* tea at different leaf loading (%); Table S3b: Analysis of variance results for leaf loading; Table S4a: Chemical component content in *Lespedeza juncea* tea at different roasting temperature (%); Table S4b: Analysis of variance results for roasting temperature; Table S5a: Chemical component content in *Lespedeza juncea* tea at different roasting time (%); Table S5b: Analysis of variance results for roasting time; Table S6: Results of orthogonal experiment for optimizing the fixation process of *Lespedeza juncea* tea; Table S7: Volatile compounds detected in *Lespedeza juncea* tea; Table S8: Overall odor activity values (OAV) of total volatile compounds in *Lespedeza juncea* tea; Table S9: Overall odor activity values (OAV) of total volatile compounds in *Lespedeza juncea* tea (continued); Table S10: Odor activity values (OAV) of volatile compounds unique to each group of *Lespedeza juncea* tea; Table S11: Chemical component content in *Lespedeza juncea* tea under different roasting condition; Table S12: Summary of major metabolites and their biological significance in Group I; Figure S1: Pearson correlation between neg QCsamples; Figure S2. Model ranking verification plots for each treatment group relative to the CK group (a)–(i); Figure S3. The Mass Spectrometry of 3-O-Acetylpinobanksin; Figure S4. The Mass Spectrometry of Hydroxygenkwanin; Figure S5. The Mass Spectrometry of Herbacetin; Figure S6. The Mass Spectrometry of Homoeriodictyol; Figure S7. The Mass Spectrometry of Velutin; Figure S8. The Mass Spectrometry of Kaempferol-4′,5,7-trimethoxy; Figure S9. The Mass Spectrometry of 3″,4″-di-O-acetylafzelin; Figure S10. The Mass Spectrometry of 5,2′,5′-Trihydroxy-3,7,8-trimethoxyflavone-2′-acetate; Figure S11. The Mass Spectrometry of Formononetin-7-O-β-D-glucuronide; Figure S12. The Mass Spectrometry of Scutellarein; Figure S13. The Mass Spectrometry of Tricin 7-(6-malonylglucoside). References [44,49,50,52,53,62,63,64,65,66,67,68,69,70,71,72,81,82,83,84,85,86,87,88,89,90,91] are cited in the Supplementary Materials.
