# Supplementary material for: Process Optimization and Flavor Analysis of Lespedeza juncea Tea Based on HS-SPME-GC-O-MS, LC-MS, and Sensory Evaluation
Source: Foods. 2026 Mar 18;15(6):1066. doi: 10.3390/foods15061066 (PMC13025511; doi:10.3390/foods15061066)
Supplement: Supplementary file 1 [file foods-15-01066-s001.zip › foods-4167660-supplementary.pdf]

Process Optimization and Flavor Analysis of *Lespedeza Juncea* Tea Based on HS-SPME-GC-O-MS, LC-MS and Sensory Evaluation

Hui Fu [1], Yuning Liu [1,*], Pengcheng Qiu [2], Ying Ying [1], Yiying Huang [1], Runhang Li [1], Qiang Yin [1, 3], Yuanfa Meng [1, 3], Zhihui Zhao [1], Xiaowei Jiang [1], Hongxin Wu [1,*]

Table S1. Sensory evaluation scoring criteria and basis for *Lespedeza juncea* tea

Item	Weight	Evaluation basis	Points
Appearance (Shape. Tenderness. Color. Integrity. Purity)	25	Compact strands, no special shaping, color uniformly tender green, emerald green, dark green, or vivid green, good purity.	90-99
		Compact buds, no special shaping, color dark green, yellow-green, or blue-green, fairly uniform, good purity.	80-89
		Compact buds, no special shaping, color dark brown, old gray, or gray-green, fairly uniform, fairly good purity.	70-79
Liquor Color (Color type. Intensity. Brightness. Clarity)	10	Bright tender green or bright green	90-99
		Fairly bright tender green or bright yellow	80-89
		Deep yellow or yellow-green, lacking brightness or cloudy	70-79
Aroma (Type. Intensity. Purity. Persistence)	25	Sweet fragrance or mellow sweetness, rich and mellow with freshness, rich and mellow with freshness	90-99
		Refreshing, moderately rich, moderately mellow	80-89
		Moderately mellow, richly astringent, greenish astringency	70-79
Taste (Intensity. Body. Mellow/Astringent. Purity/Distinctiveness. Freshness/Dullness)	30	Highly refreshing with chestnut aroma or tender fragrance or floral notes	90-99
		Fresh, fairly high and crisp, roasted aroma	80-89
		Fairly mellow, stale, over-roasted	70-79
Leaf base (Tenderness. Color. Brightness. Uniformity)	10	Bright green, tender, uniform	90-99
		Bright green, fairly uniform	80-89
		Yellowish-green, uneven	70-79

Table S2. Mass concentration of standard solution and reference solution (per 1 litre)

NO.	Solution	Composition
1	Reference solution	2.2365 g KCl + 0.045 g L+Tartaric acid
2	Salty-sample	22.365 g KCl + 0.045 g L+Tartaric acid
3	Sour-sample	2.2365 g KCl + 0.045 g L+Tartaric acid
4	Umami-sample	2.2365 g KCl + 0.045 g L+Tartaric acid + 1.691 g Sodium L-glutamate monohydrate
5	Bitter (+)-sample	2.2365 g KCl + 0.045 g L+Tartaric acid + 0.0364 g Quinine hydrochloride dihydrate
6	Bitter (-)-sample	2.2365 g KCl + 0.045 g L+Tartaric acid + 0.1mL iso-α-acid
7	Astringent-sample	2.2365 g KCl + 0.045 g L+Tartaric acid + 0.5 g Tannic acid

Note: Here is calibration protocol of E-tongue

Table S3a Chemical component content in *Lespedeza juncea* tea at different leaf loading (%)

Leaf loading (g)	Flavonoids	Tea polyphenols	Soluble sugars	Free amino acids
CK	3.55±0.25^{a}	4.48±0.08^{a}	9.54±0.18^{c}	1.42±0.04^{a}
W-25	3.69±0.29^{a}	3.72±0.11^{d}	11.71±1.67^{b}	0.40±0.03^{f}
W-50	3.72±0.31^{a}	3.89±0.14^{c}	12.00±0.73^{b}	0.42±0.02^{f}
W-75	3.73±0.23^{a}	4.20±0.06^{b}	12.67±0.76^{ab}	0.63±0.03^{e}
W-100	3.77±0.31^{a}	4.23±0.04^{b}	13.6±1.44^{ab}	0.69±0.04^{d}
W-125	3.93±0.18^{a}	4.24±0.07^{b}	14.2±1.06^{a}	0.81±0.03^{c}
W-150	3.77±0.29^{a}	4.25±0.05^{b}	13.5±0.89^{ab}	0.83±0.03^{c}
W-175	3.73±0.20^{a}	4.27±0.05^{b}	12.97±1.00^{ab}	0.92±0.04^{b}

Note: T=160°C, t=210s. Contents are presented as mean ± standard deviation (mean ± SD, n =3). Different letters indicate significant differences by Duncan's test was employed ($p < 0.05$).

Table S3b Analysis of variance results for leaves loading

Factor	Chemical component		Sum of Squares	df	Mean Square	F	Significance (P)
Leaf loading	Flavonoids	Between Groups	0.223	7	0.032	0.46	0.849
		Within Groups	1.107	16	0.069		
		Total	1.329	23			
	Tea polyphenols	Between Groups	1.195	7	0.171	17.18	< 0.01
		Within Groups	0.159	16	0.01		
		Total	1.354	23			
	Soluble sugars	Between Groups	45.265	7	6.466	5.90	0.002
		Within Groups	17.526	16	1.095		
		Total	62.792	23			
	Free amino acids	Between Groups	2.209	7	0.316	372.91	< 0.01
		Within Groups	0.014	16	0.001		
		Total	2.222	23			

Table S4a Chemical component content in *Lespedeza juncea* tea at different roasting temperature (%)

Roasting temperature (°C)	Flavonoids	Tea polyphenols	Soluble sugars	Free amino acids
CK	3.55±0.25^{bc}	4.48±0.08^{a}	9.54±0.18^{c}	1.42±0.04^{ab}
T-100	3.55±0.31^{bc}	4.31±0.22^{ab}	11.97±0.54^{ab}	1.41±0.03^{ab}
T-120	3.76±0.21^{bc}	4.26±0.10^{ab}	12.23±0.85^{ab}	1.40±0.04^{ab}
T-140	3.84±0.27^{bc}	4.26±0.23^{ab}	12.35±0.41^{ab}	1.40±0.04^{b}
T-160	3.83±0.23^{ab}	4.22±0.13^{ab}	12.52±0.68^{a}	1.22±0.07^{c}
T-180	4.13±0.20^{bc}	4.08±0.20^{b}	12.31±0.64^{ab}	1.21±0.03^{c}
T-200	4.65±0.22^{a}	4.01±0.05^{b}	10.57±0.72^{bc}	0.97±0.05^{d}
T-220	3.03±0.26^{c}	3.95±0.32^{b}	9.98±0.49^{c}	0.87±0.05^{e}

Note: W=100 g, t=210s. Contents are presented as mean ± standard deviation (mean ± SD, n =3). Different letters indicate significant differences by Duncan's test was employed ($p < 0.05$).

Table S4b Analysis of variance results for roasting temperature

Factor	Chemical component		Sum of Squares	df	Mean Square	F	Significance (P)
Roasting temperature	Flavonoids	Between Groups	4.673	7	0.668	4.83	0.004
		Within Groups	2.21	16	0.138		
		Total	6.884	23			
	Tea polyphenols	Between Groups	0.637	7	0.091	8.51	＜0.01
		Within Groups	0.171	16	0.011		
		Total	0.808	23			
	Soluble sugars	Between Groups	30.566	7	4.367	4.67	0.005
		Within Groups	14.956	16	0.935		
		Total	45.521	23			
	Free amino acids	Between Groups	0.982	7	0.14	84.21	＜0.01
		Within Groups	0.027	16	0.002		
		Total	1.009	23			

Table S5a Chemical component content in *Lespedeza juncea* tea at different roasting time (%)

Roasting time (s)	Flavonoids	Tea polyphenols	Soluble sugars	Free amino acids
CK	3.55±0.25[a]	4.48±0.08[a]	9.54±0.18[a]	1.42±0.04[a]
t-120	3.82±0.24[a]	4.01±0.27[b]	10.04±0.52[a]	1.39±0.04[ab]
t-150	4.51±0.10[a]	3.86±0.14[b]	11.43±0.63[a]	1.37±0.05[ab]
t-180	4.46±0.11[a]	3.78±0.29[b]	11.62±0.40[a]	1.32±0.05[b]
t-210	4.07±0.35[a]	3.71±0.22[b]	11.43±0.55[a]	1.23±0.06[c]
t-240	3.98±0.23[a]	3.65±0.25[b]	11.26±0.19[a]	1.11±0.05[d]
t-270	3.95±0.36[a]	3.59±0.15[b]	11.23±0.67[a]	1.01±0.04[de]
t-300	3.78±0.28[a]	3.56±0.28[b]	10.72±0.10[a]	0.93±0.04[e]

Note: W=100 g, T=160°C. Contents are presented as mean ± standard deviation (mean ± SD, n =3). Different letters indicate significant differences by Duncan's test was employed ($p < 0.05$).

Table S5b Analysis of variance results for roasting time

Factor	Chemical component		Sum of Squares	df	Mean Square	F	Significance (P)
Roasting time	Flavonoids	Between Groups	2.271	7	0.324	2.46	0.064
		Within Groups	2.104	16	0.132		
		Total	4.375	23			
	Tea polyphenols	Between Groups	1.879	7	0.268	4.09	0.009
		Within Groups	1.049	16	0.066		
		Total	2.928	23			
	Soluble sugars	Between Groups	11.86	7	1.694	6.22	0.001
		Within Groups	4.357	16	0.272		
		Total	16.217	23			

Free amino acids	Between Groups	0.732	7	0.105	47.47	＜0.01
	Within Groups	0.035	16	0.002		
	Total	0.767	23			

Table S6. Results of orthogonal experiment for optimizing the fixation process of *Lespedeza juncea* tea

**NO.**	**W**	**T**	**t**	**Flavonoids (%)**
1	125	200	210	3.67
2	175	160	180	3.84
3	175	200	150	3.95
4	150	160	210	3.71
5	175	180	210	3.69
6	150	200	180	4.11
7	125	180	180	3.73
8	150	180	150	3.69
9	125	160	150	3.77
K1	11.17	11.33	11.41	
K2	11.51	11.11	11.68	
K3	11.49	11.73	11.07	
**k1**	3.72	3.78	3.80	
**k2**	3.84	3.70	3.89	
**k3**	3.83	3.91	3.69	
R	0.11	0.21	0.20	
Primary factors	3	1	2	
Optimal level	2	3	2	
Optimal solution	$W_2T_3t_2$			

Table S7. Volatile compounds detected in *Lespedeza juncea* tea

No.	RT (min)	Class/Volatile compounds	CAS No.	Content (μg/g)										Aroma description
				CK	A	B	C	D	E	G	H	I	K	
		**Alcohols**												
**1**	4.70	2-Methyl-1-Butanol	137-32-6	— —	— —	— —	— —	— —	— —	— —	— —	1.88	— —	A faint hint of alcohol, grass, and cocoa
**2**	7.79	1-Hexanol	111-27-3	— —	— —	— —	11.34	— —	— —	14.88	3.67	5.35	3.52	Herbal notes, fresh fermented fruit aroma, citrus peel scent
**3**	10.59	1-Octen-3-ol	3391-86-4	— —	229.84	303.66	190.22	253.31	318.70	304.70	336.93	365.49	338.34	The earthy scent of soil, the earthy aroma of mushrooms, the earthy flavor of vegetables, the savory richness of broth.
**4**	11.72	2-Ethylhexanol	104-76-7	— —	1.11	3.14	2.70	1.37	2.29	3.49	3.14	5.24	— —	Citrus aroma, buttery flavor
**5**	12.55	*(E)*-2-Octen-1-ol	18409-17-1	— —	5.20	— —	4.28	4.28	— —	— —	— —	8.83	— —	Green notes (sweet, citrusy), fatty aroma profile (buttery, fruity)
**6**	12.62	1-Octanol	111-87-5	— —	15.16	20.87	17.79	16.74	25.40	21.60	25.41	31.87	18.43	Waxy flavor, floral aroma, sweetness, fatty taste, coconut flavor
**7**	13.287	Linalool	78-70-6	— —	— —	— —	— —	— —	— —	— —	— —	8.83	— —	Floral aroma, citrus flavor
**8**	14.78	1-Nonanol	143-08-8	— —	0.87	1.83	1.34	0.77	1.22	1.34	1.57	6.01	1.63	Floral aroma (fresh floral notes, earthy undertones); Waxy flavor (aldehyde waxiness, citrus oil, spicy fatty notes)
**9**	20.28	1-Dodecanol	112-53-8	— —	5.20	10.39	10.51	4.28	7.45	9.04	6.03	16.72	18.29	Wax aroma (earthy, soapy, honeyed, coconut); Soap flavor (soapy, waxy, aldehydic, fatty)

**10**	22.64	Cedrol	77-53-2	— —	— —	3.49	3.11	— —	6.43	4.06	2.88	9.03	5.01	Woody aroma (cedarwood, sweet); Woody flavor (amber floral, cypress, musky)
		**Aldehydes**												
**11**	7.41	Hex-2-enal	505-57-7	— —	1.01	7.25	3.22	— —	1.97	2.94	5.76	8.86	11.65	Fresh taste, scent of fresh leaves, herbal and fruity notes
**12**	8.70	Heptanal	111-71-7	— —	5.44	10.31	— —	6.38	5.98	— —	10.74	14.27	11.79	Fresh taste, scent of fresh leaves, herbal and fruity notes
**13**	11.19	Octanal	124-13-0	13.42	7.92	11.09	13.71	9.36	9.80	15.70	9.96	16.20	14.63	Aldehyde aromas and flavors; (lime, citrus, orange peel, herbs, fatty notes)
**14**	13.510	Nonanal	124-19-6	94.57	31.13	87.16	45.50	44.01	38.24	53.82	73.36	72.52	57.86	Orange, rose, fresh, iris, creamy
**15**	15.50	Decanal	112-31-2	24.92	2.80	5.15	3.52	3.52	3.73	3.60	4.72	6.11	8.27	Sweetness, peeled orange, citrus tree, floral fragrance
**16**	18.06	γ-Nonanolactone	104-61-0	— —	— —	26.33	— —	— —	— —	22.59	— —	— —	— —	Coconut scent
**17**	19.26	Dodecanal	112-54-9	— —	1.60	2.18	1.43	1.37	— —	1.46	— —	1.38	2.17	Aldehyde aromas (soap, wax, citrus, orange peel, floral); Soap flavor profile (soap, citrus, orange peel)
**18**	22.30	Tetradecanal	124-25-4	80.51	— —	1.05	— —	— —	0.74	— —	— —	— —	1.07	Fat, cured meats, dairy products, buttery flavor, fishy odor, fruity notes, pear flavor
		**Ketones**												
**19**	20.47	β-Ionone	79-77-6	— —	1.65	2.79	1.26	1.93	1.42	2.62	7.85	8.38	5.42	Floral aromas (sweet, berry, fruity) Woody flavors (oaky, berry, floral, unripe fruit)

**20**	25.16	Phytone	502-69-2	— —	— —	— —	1.86	— —	— —	— —	— —	— —	— —	Floral aroma (herbal woody notes) Unripe flavor (fruity notes of watermelon and kiwi)
		**Esters**												
**21**	10.90	Propyl valerate	141-06-0	— —	4.45	— —	— —	3.17	4.43	3.41	— —	— —	— —	Faint fruit, pineapple, and animal notes
**22**	15.20	Amyl valerate	2173-56-0	— —	2.47	1.66	2.18	2.42	2.23	1.49	1.57	0.83	1.63	Fruity aroma (ripe apple scent)
**23**	16.03	*(Z)*-3-Hexenyl 2-methylbutanoate	53398-85-9	— —	3.97	— —	— —	— —	— —	1.82	— —	3.59	1.36	Green notes and flavors (fresh apple, pear, and berry notes, tartness)
**24**	16.15	*(Z)*-3-Hexenyl 3-methylbutanoate	35154-45-1	— —	— —	3.93	— —	— —	— —	4.14	2.88	— —	6.37	Green notes and flavors (buttery, floral, fruity, earthy)
**25**	17.99	Isobutyl benzoate	120-50-3	— —	— —	1.14	0.99	0.75	— —	1.46	1.31	2.56	1.63	Balsamic aroma (intense fruitiness, musty notes); fruity flavor (fruity, cherry notes)
**26**	23.64	2-Ethylhexyl benzoate	5444-75-7	— —	1.82	— —	2.45	— —	2.27	1.16	— —	3.05	2.17	A delicate, subtle fragrance with a faint aroma.
**27**	26.09	Methyl palmitate	112-39-0	— —	1.01	— —	1.00	1.37	— —	— —	— —	0.83	— —	Waxy taste (fatty taste)
		**Others**												
**28**	10.78	*β*-Pinene	127-91-3	— —	— —	— —	— —	— —	— —	— —	— —	1.57	— —	Herbal scent (dry wood, hay aroma); Pine flavor (fresh pine scent, mildly pungent taste)
**29**	16.80	Nonanoic acid	112-05-0	— —	— —	— —	— —	— —	— —	— —	— —	29.52	— —	Waxy aroma; buttery flavor
**30**	20.01	*(E)*-*β*-Farnesene	18794-84-8	— —	— —	— —	— —	— —	— —	— —	— —	— —	0.95	Woody, citrus, herbal, sweet
**31**	21.64	*β*-Caryophyllene	87-44-5	— —	— —	— —	— —	— —	— —	— —	1.05	— —	— —	Sweetness, woody notes, nutty husk flavor

Note: A (160°C, 150s, 125 g), B (160°C, 180s, 175 g), C (160°C, 210s, 150 g), D (180°C, 150s, 150 g), E (180°C, 180s, 125 g), G (180°C, 210s, 175 g), H (200°C, 150s,175 g), I (200°C,

180s, 150 g), K (200°C, 210s, 125 g), CK (air dried); The odor descriptions of volatile compounds are sourced from www.perflavory.com

Table S8. Overall odor activity values (OAV) of total volatile compounds in *Lespedeza juncea* tea

No.	RT (min)	Volatile compounds	CAS No.	OT (μg/L)	Concentration and odor activity value (OAV)									
					A		B		C		D		E	
					(μg/L)	OAV	(μg/L)	OAV	(μg/L)	OAV	(μg/L)	OAV	(μg/L)	OAV
1	10.59	1-Octen-3-ol	3391-86-4	1[1]	46.03	46.03	47.98	47.98	38.01	38.01	50.63	50.63	64.38	64.38
2	12.62	1-Octanol	111-87-5	3[1]	3.04	1.01	4.19	1.40	3.56	1.19	3.34	1.12	5.08	1.69
3	14.78	1-Nonanol	143-08-8	0.9[1]	0.175	0.19	0.39	0.43	0.27	0.30	0.15	0.17	0.24	0.27
4	20.28	1-Dodecanol	112-53-8	66[2]	1.04	0.016	2.10	0.032	2.10	0.032	1.13	0.017	1.50	0.023
5	11.19	Octanal	124-13-0	0.01[1]	1.59	158.67	2.22	222.36	3.06	306.36	1.87	187.05	1.98	198
6	13.51	1-Nonanal	124-19-6	1.1[1]	6.23	5.67	17.45	15.87	9.11	8.28	8.7	8.00	7.72	7.02
7	15.50	Decanal	112-31-2	0.4[1]	0.56	1.40	1.03	2.58	0.71	1.76	0.70	1.76	0.75	1.88
8	20.47	*β*-Ionone	79-77-6	0.007[3]	0.33	47.32	0.56	80.19	0.25	35.98	0.38	55.12	0.29	40.91
9	15.20	Amyl valerate	2173-56-0	NF	0.0025	——	0.0017	——	0.0029	——	0.0024	——	0.0022	——

Note: "NF" indicates no relevant reference threshold found

"——" indicates no data available

Table S9. Overall odor activity values (OAV) of total volatile compounds in *Lespedeza juncea* tea (continued)

No.	RT(min)	Volatile compounds	CAS No.	OT (μg/L)	Concentration and odor activity value (OAV)							
					G		H		I		K	
					(μg/L)	OAV	(μg/L)	OAV	(μg/L)	OAV	(μg/L)	OAV
1	10.59	1-Octen-3-ol	3391-86-4	1[1]	60.94	60.94	67.39	67.39	73.04	73.04	71.44	71.44
2	12.62	1-Octanol	111-87-5	3[1]	4.32	1.44	5.08	1.69	6.37	2.12	4.00	1.33
3	14.78	1-Nonanol	143-08-8	0.9[1]	0.27	**0.30**	0.31	**0.35**	0.35	**0.39**	0.34	**0.38**
4	20.28	1-Dodecanol	112-53-8	66[2]	1.81	**0.027**	1.21	**0.018**	3.34	**0.051**	4.77	**0.072**
5	11.19	Octanal	124-13-0	0.01[1]	3.14	313.91	1.99	199.12	3.24	323.72	3.09	308.92
6	13.51	1-Nonanal	124-19-6	1.1[1]	10.76	9.79	14.67	13.34	14.49	13.17	12.20	11.09
7	15.50	Decanal	112-31-2	0.4[1]	0.72	1.80	0.94	2.36	1.22	3.05	1.72	4.29
8	20.47	*β*-Ionone	79-77-6	0.007[3]	0.52	**74.82**	1.57	**223.99**	1.68	**239.54**	1.14	**162.43**
9	15.20	Amyl valerate	2173-56-0	NF	0.0015	——	0.0016	——	0.00083	——	0.0016	——

Note: "NF" indicates no relevant reference threshold found

"——" indicates no data available

Table S10. Odor activity values (OAV) of volatile compounds unique to each group of *Lespedeza juncea* tea

No.	RT(min)	Volatile compounds	CAS No.	OT (μg/L)	OAV	Concentration (μg/L)			
						C	H	I	K
1	4.70	2-Methyl-1-butanol	137-32-6	NF	——	——	——	0.377	——
2	13.28	Linalool	78-70-6	0.22[4]	9.0	——	——	1.76	——
3	25.16	6,10,14-trimethyl-2-pentad ecanone	502-69-2	NF	——	0.0018	——	——	——
4	10.78	*β*-Pinene	127-91-3	140[5]	0.002	——	——	0.31	——
5	16.80	Nonanoic acid	112-05-0	1.5[1]	4.0	——	——	5.90	——
6	20.01	*(E)*-*β*-Farnesene	18794-8 4-8	87[4]	0.0023	——	——	——	0.20
7	21.64	*β*-Caryophyllene	87-44-5	55[5]	0.004	——	0.21	——	——

Note: "NF" indicates no relevant reference threshold found

"——" indicates no data available

Table S11. Chemical component content in *Lespedeza juncea* tea under different roasting condition

Group	Roasting conditions	Flavonoids (%)	Tea polyphenols (%)	Soluble sugars (%)	Free amino acids (%)	PAR	Aqueous extract (%)
A	160°C, 150s, 125 g	3.77±0.05[cd]	4.27±0.07[a]	12.42±0.85[cd]	1.45±0.06[a]	2.94	28.81±0.78[bc]
B	160°C, 180s, 175 g	3.84±0.05[bc]	4.18±0.03[ab]	15.46±0.79[ab]	1.45±0.03[a]	2.89	28.33±0.68[bc]
C	160°C, 210s, 150 g	3.71±0.07[cd]	4.09±0.03[b]	16.29±0.66[a]	1.43±0.01[a]	2.86	29.53±0.50[b]
D	180°C, 150s, 150 g	3.69±0.04[d]	3.95±0.03[c]	16.15±1.07[ab]	1.43±0.01[ab]	2.77	28.48±0.56[bc]
E	180°C, 180s, 125 g	3.73±0.03[cd]	3.84±0.06[cd]	14.29±0.66[bc]	1.42±0.05[ab]	2.71	28.51±0.49[bc]
G	180°C, 210s, 175 g	3.69±0.17[d]	3.81±0.10[cd]	12.92±0.94[cd]	1.40±0.03[ab]	2.72	29.40±1.24[b]
H	200°C, 150s, 175 g	3.95±0.06[b]	3.70±0.12[de]	12.78±0.72[cd]	1.36±0.01[bc]	2.73	31.29±0.45[a]
I	200°C, 180s, 150 g	4.11±0.05[a]	3.64±0.06e	12.67±0.39[cd]	1.31±0.01[c]	2.78	30.86±1.06[a]
K	200°C, 210s, 125 g	3.67±0.09[d]	3.65±0.13e	11.21±0.77[d]	1.18±0.07[d]	3.08	27.71±0.92[c]

Note: Contents are presented as mean ± standard deviation (mean ± SD, n =3). Different letters indicate significant differences among samples ($p < 0.05$).

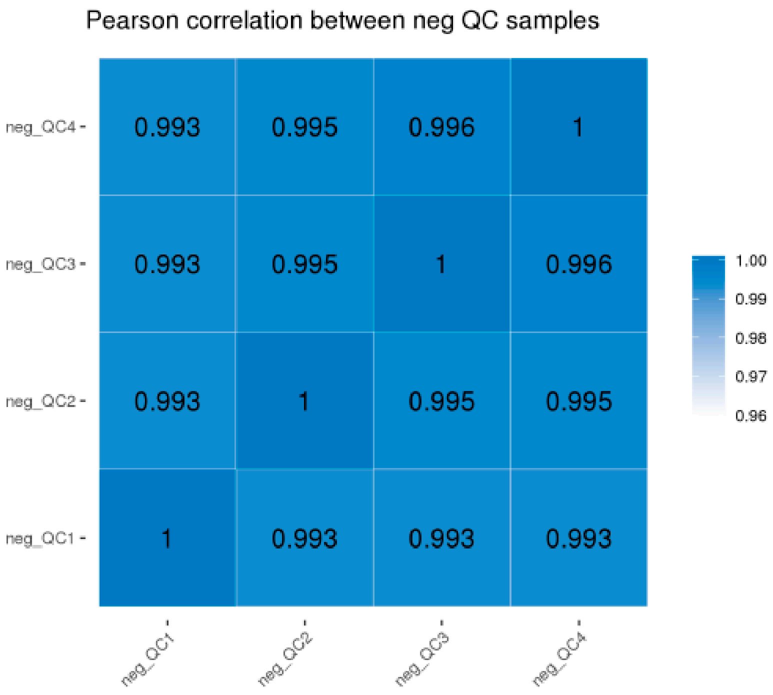

Figure S1. Pearson correlation between neg QCsamples

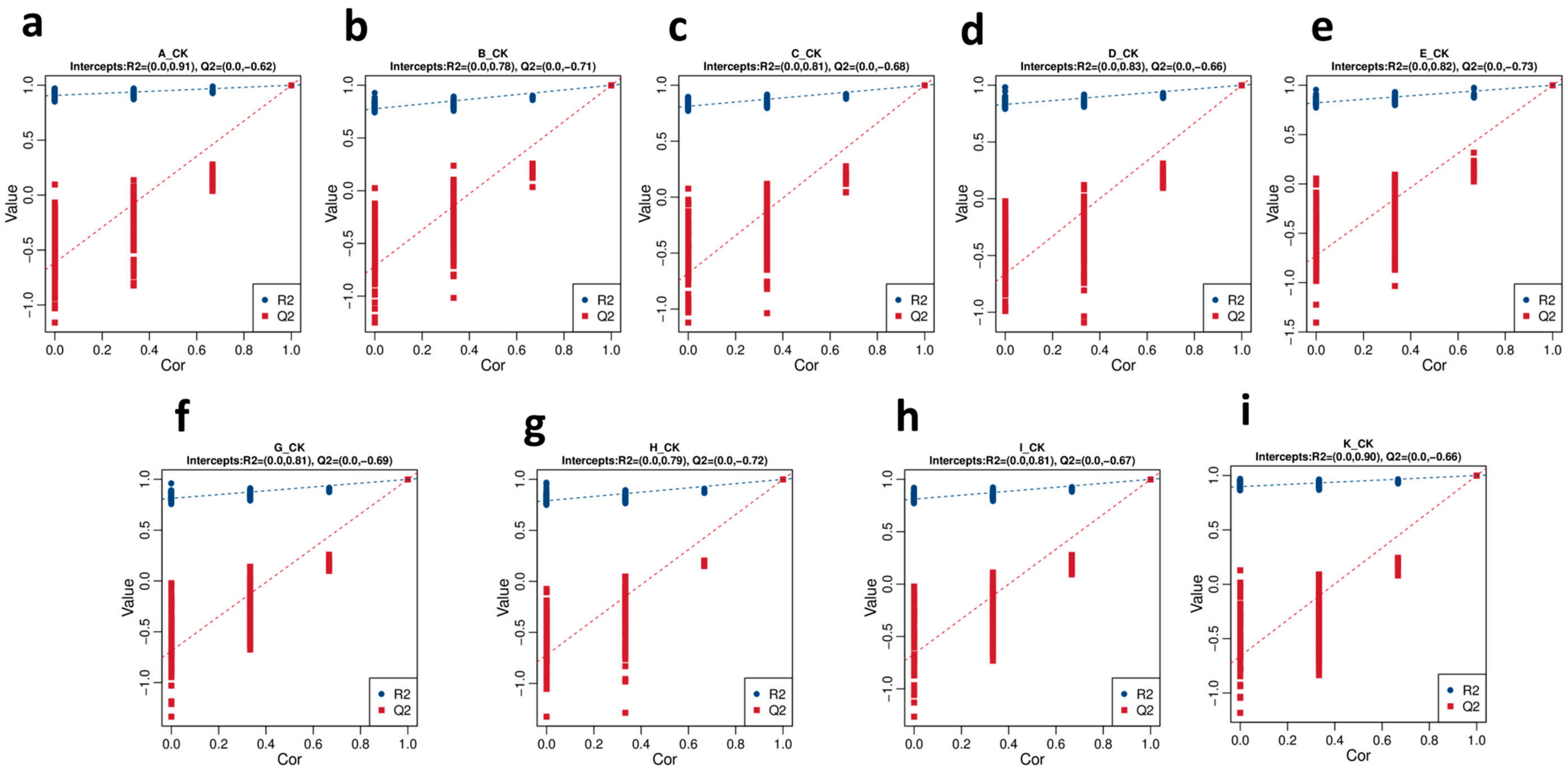

Figure S2. Model ranking verification plots for each treatment group relative to the CK group (a) - (i)

(Note: $R^2 > Q^2$ and $Q^2 < 0$ indicate that the models are not overfitted and can adequately describe the samples)

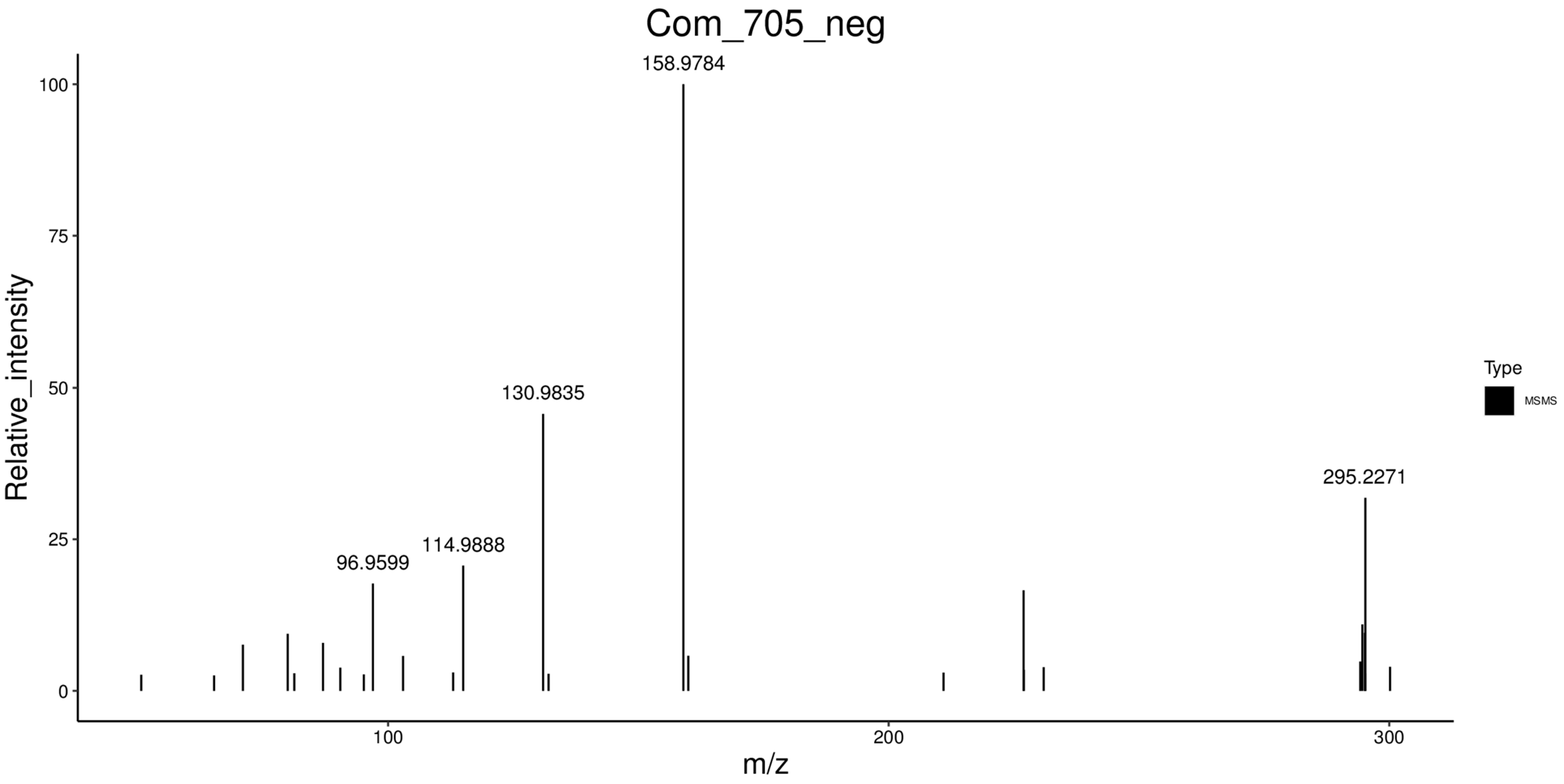

Figure S3. The Mass Spectrometry of 3-*O*-Acetylpinobanksin

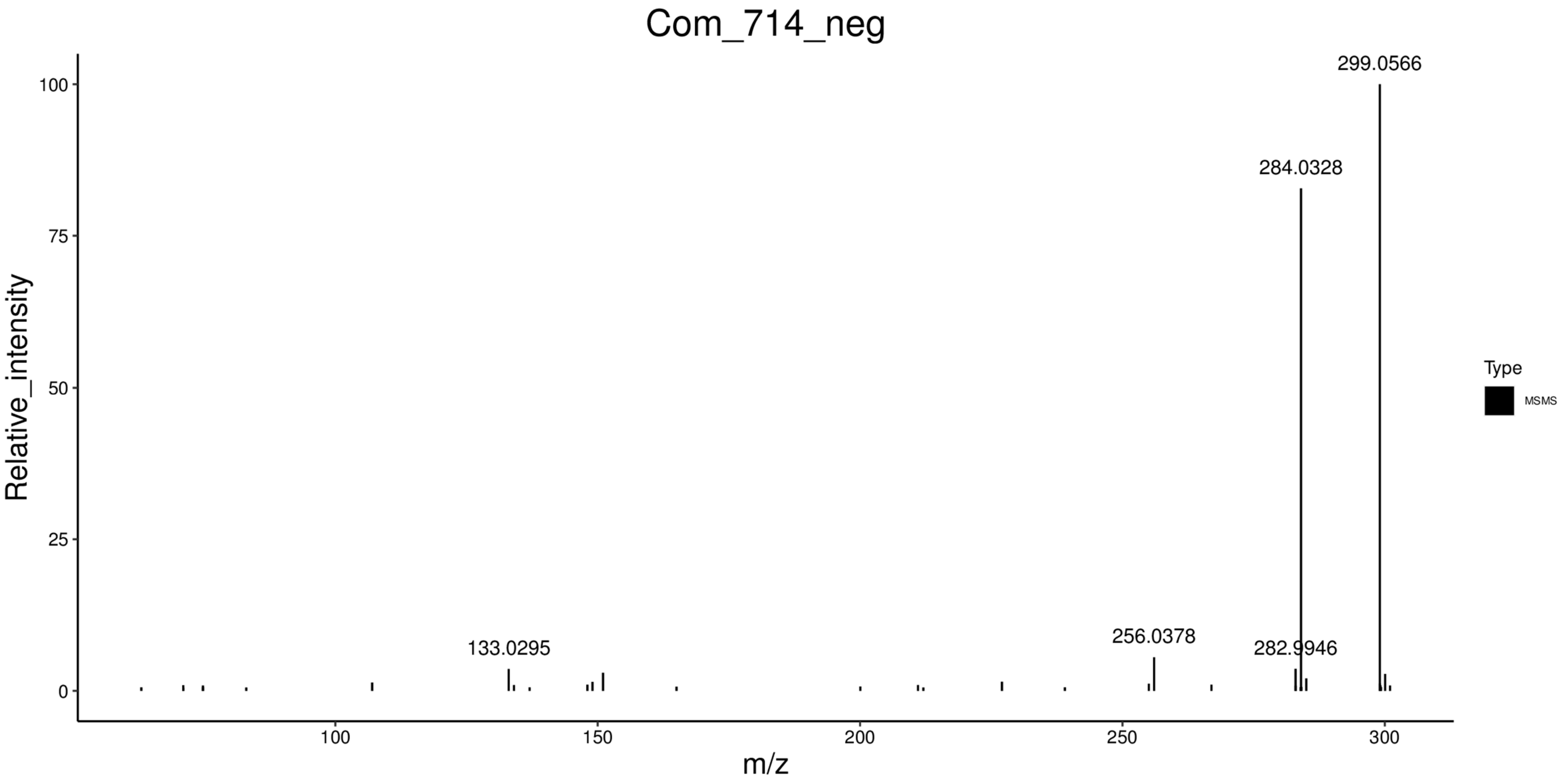

Figure S4. The Mass Spectrometry of Hydroxygenkwanin

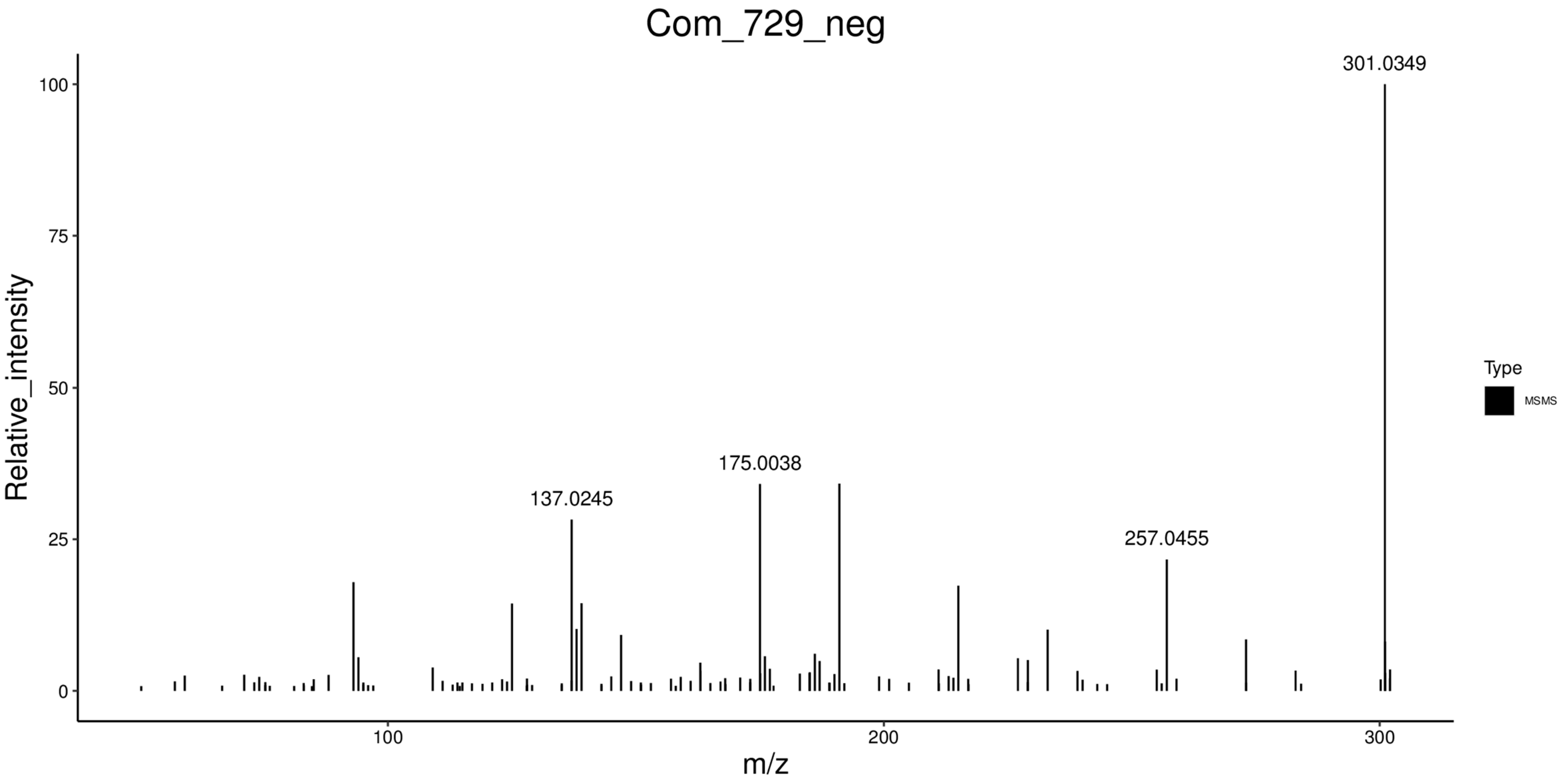

Figure S5. The Mass Spectrometry of Herbacetin

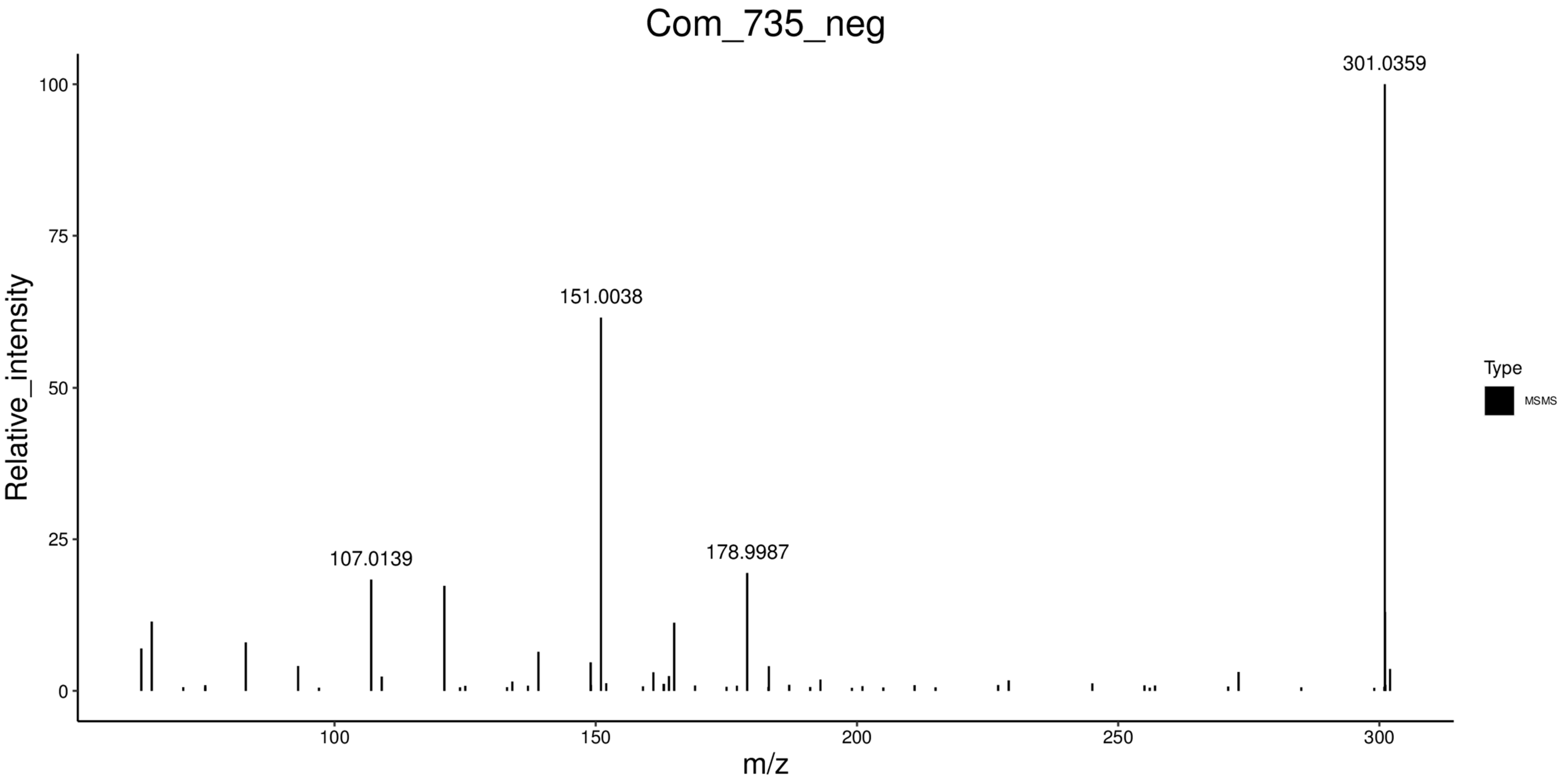

Figure S6. The Mass Spectrometry of Homoeriodictyol

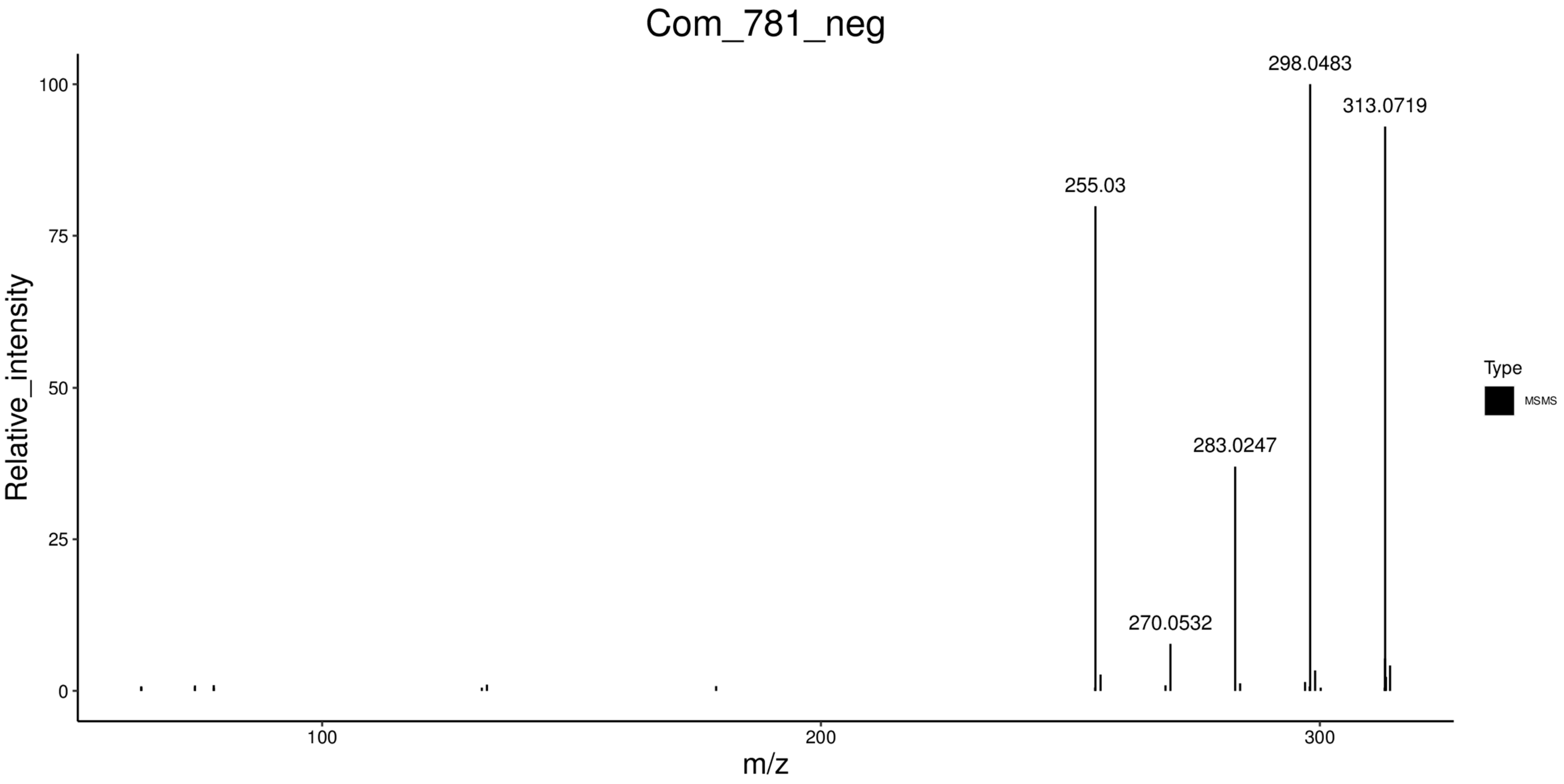

Figure S7. The Mass Spectrometry of Velutin

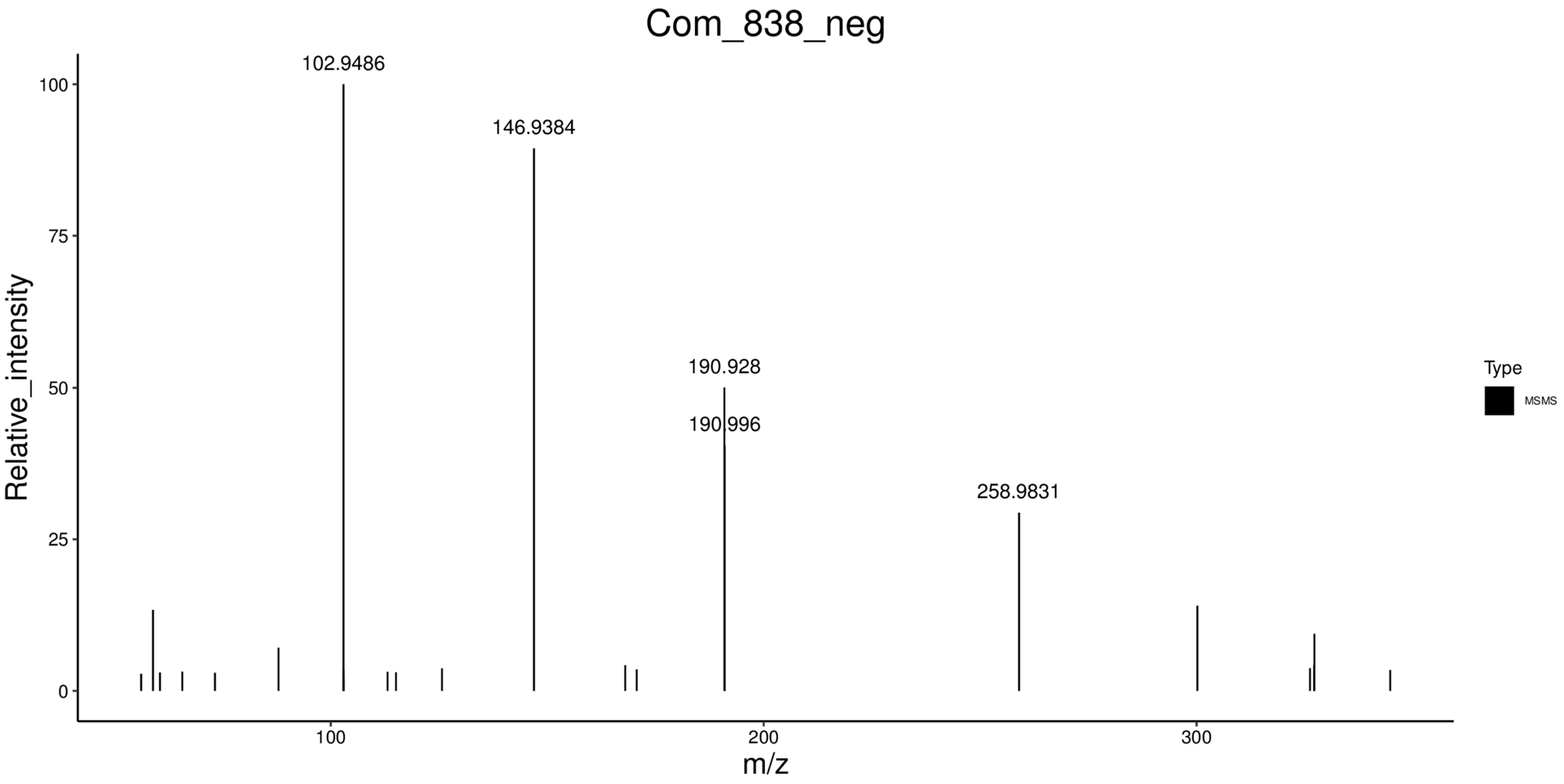

Figure S8. The Mass Spectrometry of Kaempferol-4',5,7-trimethoxy

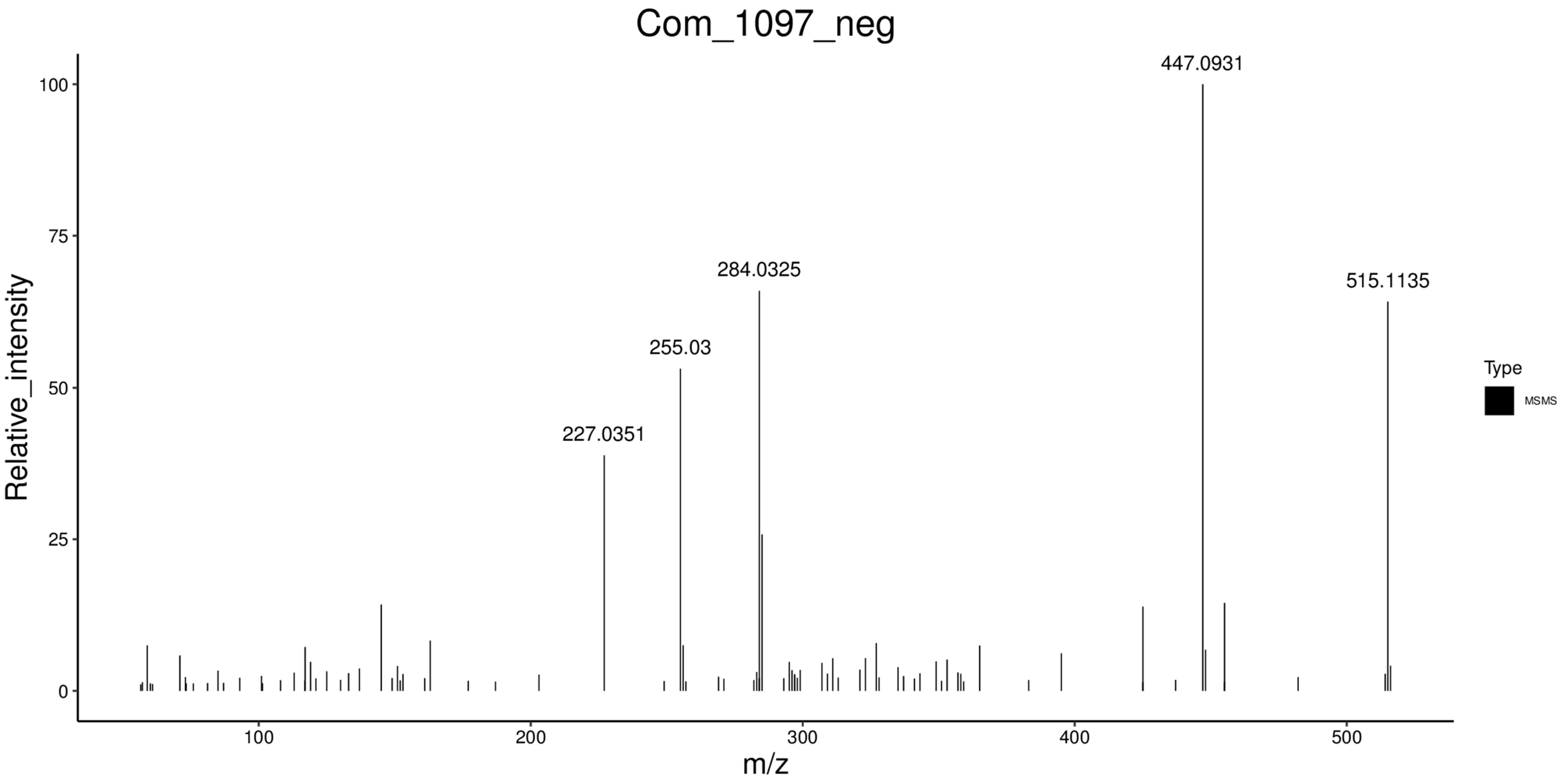

Figure S9. The Mass Spectrometry of 3'',4''-di-*O*-acetylafzelin

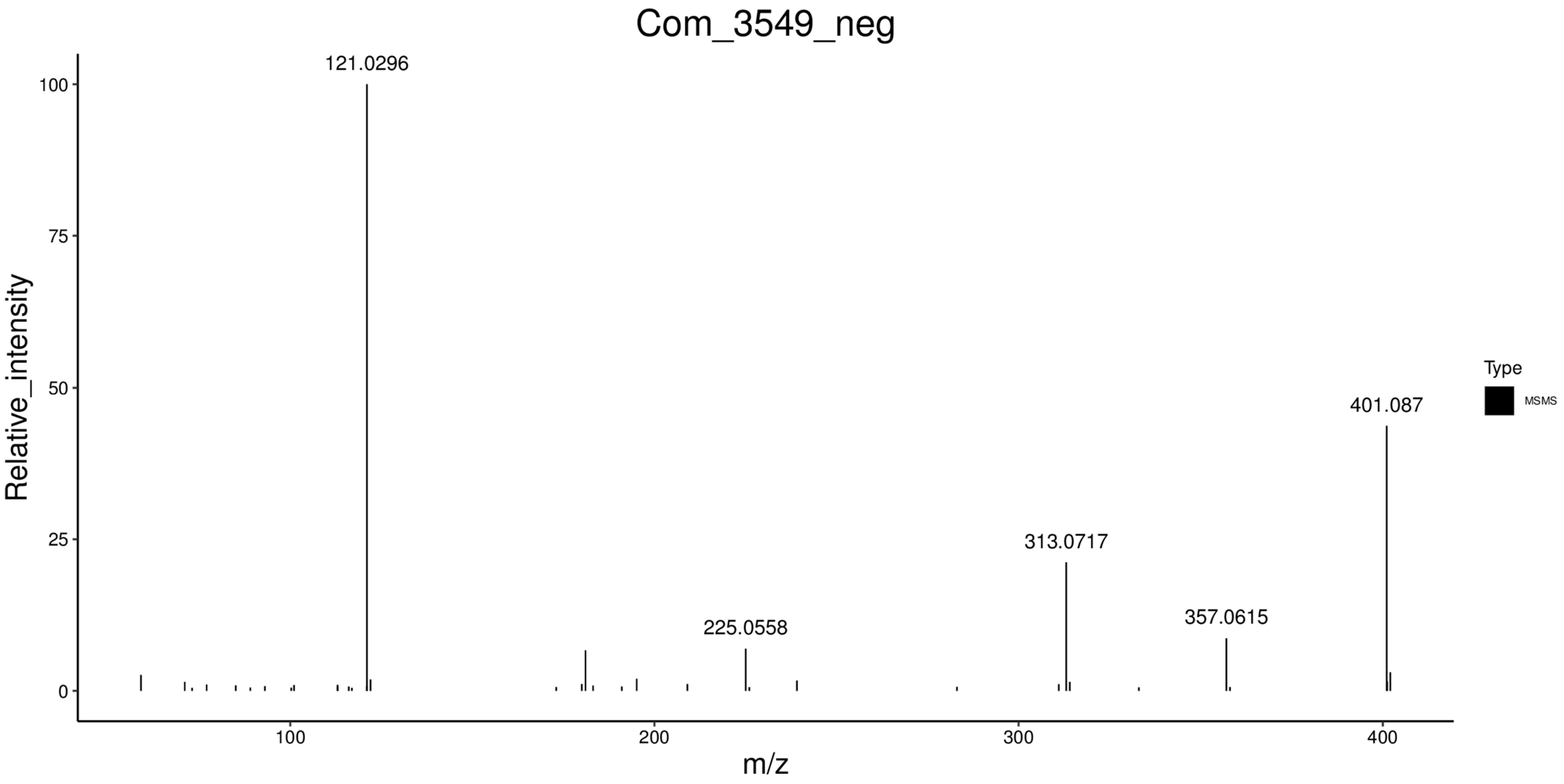

Figure S10. The Mass Spectrometry of 5,2',5'-Trihydroxy-3,7,8-trimethoxyflavone-2'-acetate

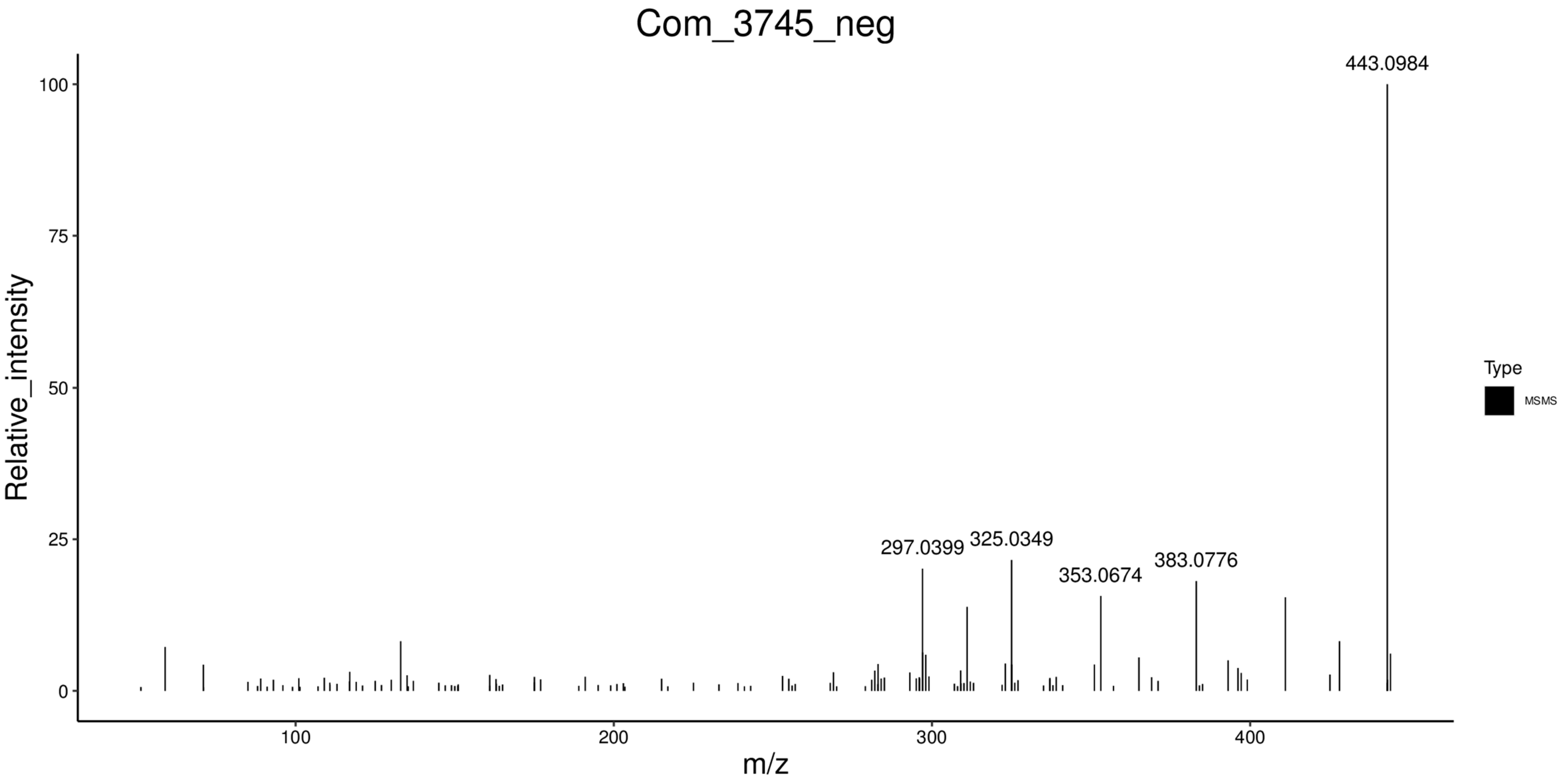

Figure S11. The Mass Spectrometry of Formononetin-7-*O*-*β*-D-glucuronide

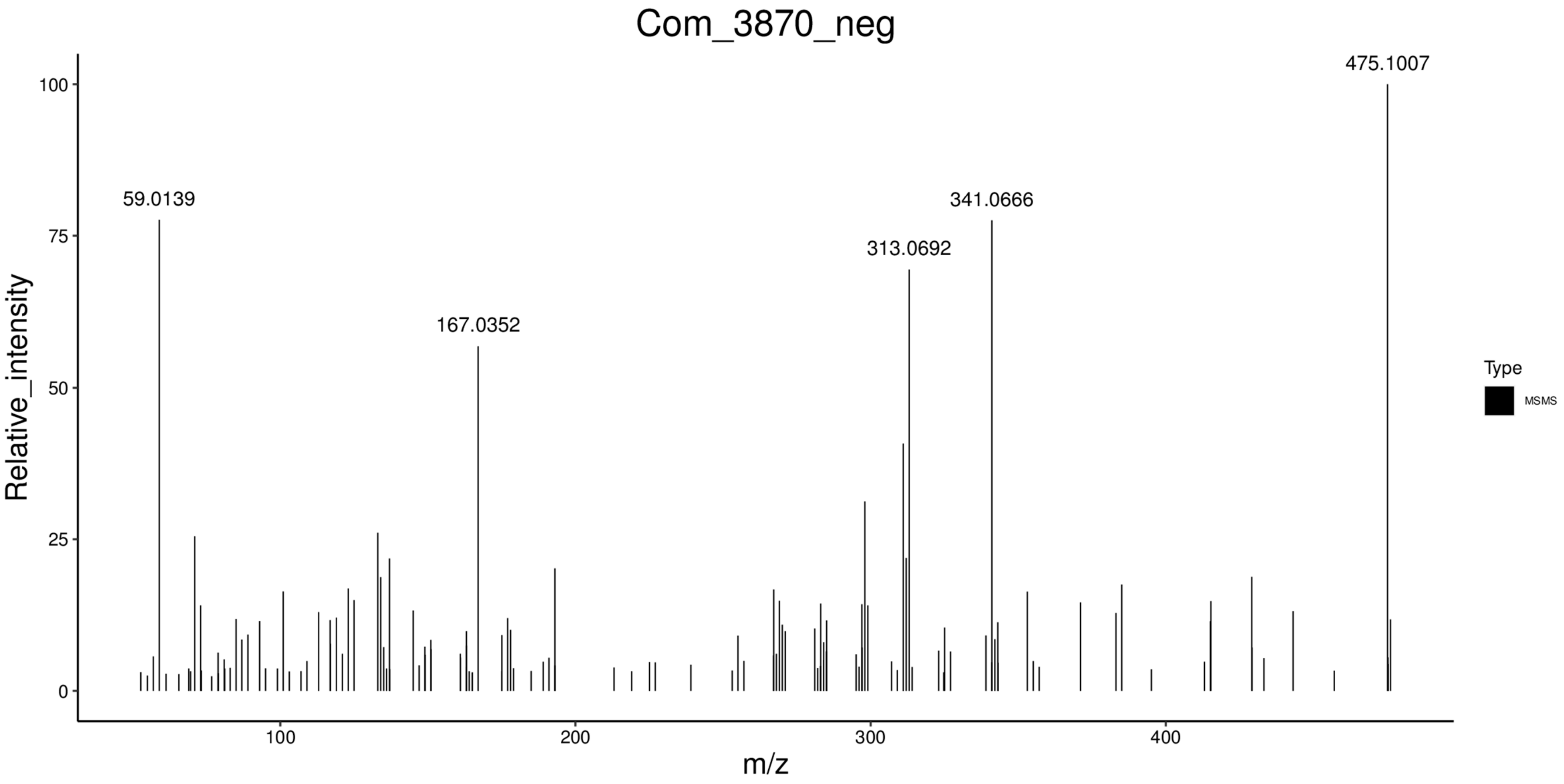

Figure S12. The Mass Spectrometry of Scutellarein

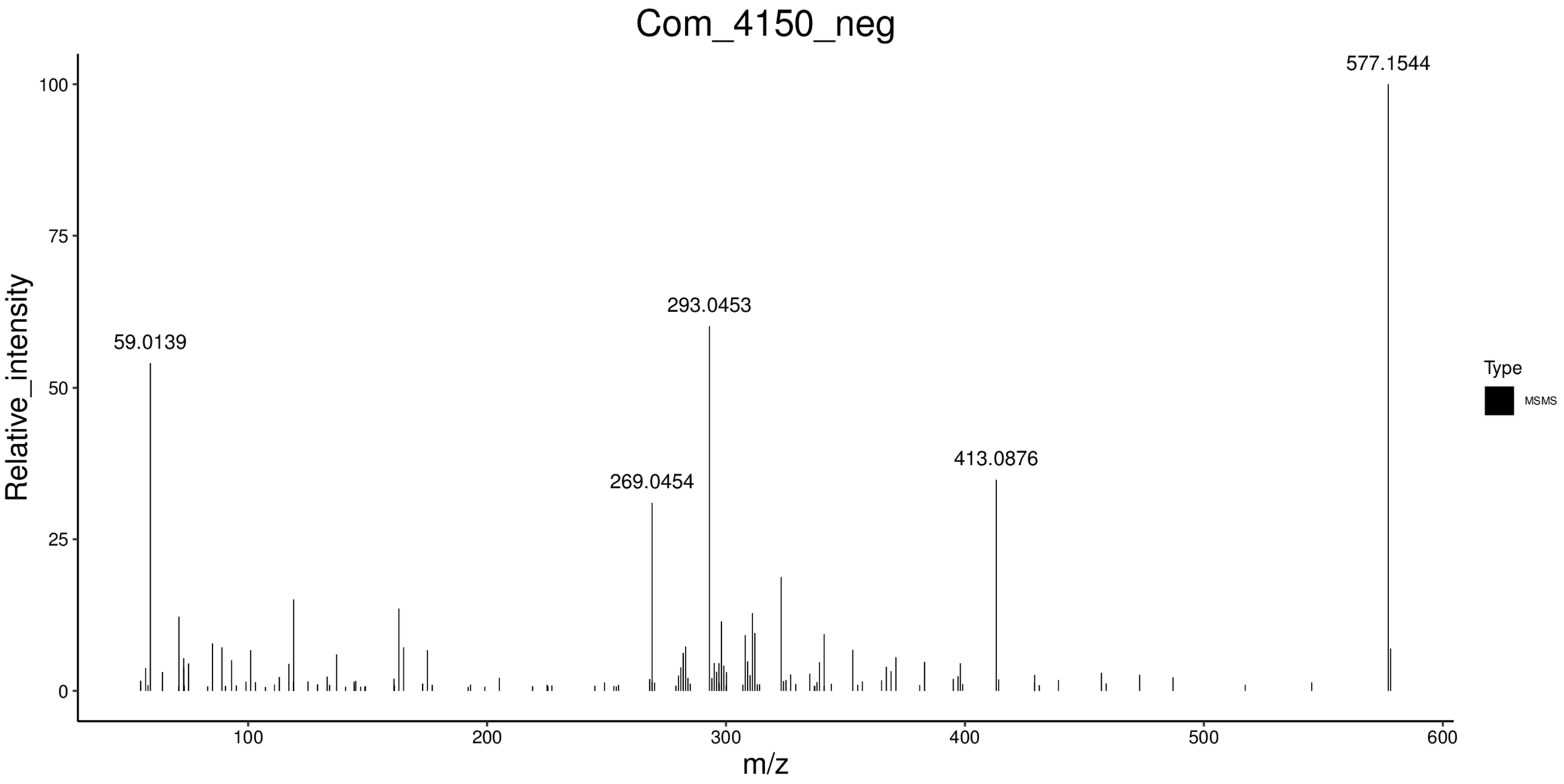

Figure S13. The Mass Spectrometry of Tricin 7-(6-malonylglucoside)

Table S12. Summary of major metabolites and their biological significance in Group I

No.	Name	Class	Level	Score	Mass Error	**CAS No.**	Biological significance
1	3'',4''-di-O-acetylafzelin	Flavonoid glycosides	1	0.56	1.07	77307-50-7	3',4''-di-O-acetylafzelin selectively inhibits the activity of ribosomal S6 kinase (RSK), and its derivatives exhibit excellent selective inhibitory effects on RSK, thereby emerging as a potential natural compound for the control and treatment of cancer.[6-8]
2	Formononetin-7-*O*-*β*-*D*-glucuronide	Flavonoid glycosides	3	——	1.33	18524-03-3	Formononetin-7-O-β-D-glucuronide is an important isoflavone aglycone metabolite in Astragali Radix (Astragalus root), serving as one of the analytes in pharmacokinetic studies[9].
3	Scutellarein	Flavonoid glycosides	2	0.57	1.23	27740-01-8	Scutellarin inhibits the proliferation, migration and clonogenic capacity of ovarian cancer cells by targeting METTL5[10].<br>Scutellarin prevents obesity-induced renal fibrosis by reducing the activation of AP-1 (composed of FOS and JUN)[11].<br>Scutellarin induces apoptosis and autophagy via the ERK1/2 and AKT signalling pathways[12].<br>Scutellarin ameliorates disease through the PPARγ/PGC-1α-Nrf2 pathway[13].
4	Tricin 7-(6-malonylglucoside)	Flavonoid glycosides	3	——	0.82	(PubChem CID : 122391240)	Tricin 7-O-β-D-glucopyranoside, the precursor compound of tricin 7-(6-malonylglucoside), is abundant in rice grains[14]. However, research on the compound tricin 7-(6-malonylglucoside) is scarce, with no relevant reports currently identified.
5	Velutin	O-methylated Flavonoids	1	0.98	1.24	25739-41-7	Velutin is a natural flavonoid compound extracted from various plants. Velutin is a flavonoid compound extracted from various plants[15]. Velutin effectively suppresses the production of inflammatory mediators induced by interleukin-1β (such as iNOS and COX-2) in nucleus pulposus cells. In a

							mouse model of disc degeneration, intraperitoneal injection of Velutin increased nucleus pulposus tissue volume, reduced histological scores, and decreased COX-2 expression, indicating its in vivo protective effects[16]. In inhibiting lipopolysaccharide-induced TNF-α and IL-6 production in macrophages, Velutin demonstrated the strongest efficacy compared to Luteolin, Apigenin, and Chrysoriol[17].
6	Homoeriodictyol	O-methylated Flavonoids	1	0.73	0.42	446-71-9	Homoeriodictyol is a dihydroflavonoid compound widely present in numerous plant species. Research indicates that this substance, along with Eriodictyol, can traverse the blood-brain barrier, ameliorating memory impairment and cognitive dysfunction induced by $A\beta_{25\text{-}35}$ in mice. It represents a potential therapeutic agent for Alzheimer's disease[18]. This compound promotes glucose uptake in differentiated Caco-2 cells by targeting the sodium-coupled glucose transporter SGLT-1, whilst simultaneously reducing serotonin release, thereby regulating glucose metabolism[19].
7	Hydroxygenkwanin	O-methylated Flavonoids	1	0.97	0.92	20243-59-8	Hydroxygenkwanin (HGK) is a natural flavonoid compound. HGK may regulate macrophage polarisation by activating the p-STAT5 and p-NF-κB signalling pathways in M1 macrophages, whilst inhibiting p-STAT6, JMJD3, and PPARγ expression in M2 macrophages. PPARγ expression in M2 macrophages. Consequently, HGK represents a natural candidate compound that inhibits peritoneal metastasis in colorectal cancer by remodelling tumour-associated macrophage polarisation[20]
8	5,2',5'-Trihydroxy-3,7,8-trimethoxyflavone-2'-acetate	O-methylated Flavonoids	3	——	1.32	PubChem CID : 44259911	5,2',5'-Trihydroxy-3,7,8-trimethoxyflavone-2'-acetate is a natural plant flavonoid extracted from ferns, belonging to the trihydroxy trimethoxyflavone class of compounds. At present, its biological significance remains unclear and warrants further investigation[21].

9	Kaempferol-4',5,7-trimethoxy	Flavones	1	0.84	0.65	1098-92-6	Kaempferol-4',5,7-trimethoxy is a derivative of kaempferol. Research indicates that kaempferol, as a common flavonoid belonging to the flavonol subclass[22] , may serve as a BKCa channel activator and Cav1.2 channel inhibitor, demonstrating potential benefits in the prevention and treatment of atherosclerosis[23]. However, no relevant studies have yet been identified concerning the compound Kaempferol-4',5,7-trimethoxy.
10	Herbacetin	Flavones	1	0.73	1.07	527-95-7	Herbacetin (HBT) is a natural flavonoid compound exhibiting antiviral, anti-fibrotic, anti-ferroptotic, and cardioprotective functions. It targets the neuraminidase (NA) protein of influenza viruses, reducing viral infection by inhibiting NA enzymatic activity. Concurrently, it suppresses the TGF-β/Smad3 signalling pathway activated by IAV, thereby diminishing expression of downstream fibrosis-associated proteins such as Fn and Snail and slowing the progression of pulmonary fibrosis[24]. Herbacetin has also been found to inhibit the activity of dengue virus NS5 methyltransferase, exerting its antiviral effect by binding near the Cap 0-RNA site and interfering with GTP binding[25]. Herbacetin mitigates ferroptosis in neurons following traumatic brain injury (TBI) by upregulating hypoxia-inducible factor-1α (Hif-1α) expression. This subsequently promotes the expression of solute carrier family 7 member 11 (SLC7A11) and glutathione peroxidase 4 (GPX4), thereby inhibiting ferroptosis[26].
11	3-*O*-Acetylpinobanksin	Flavans	1	0.97	0.25	52117-69-8	3-O-Acetylpinobanksin is a component within the flavonoid compound family. Research on the leaves of the long-stamened stone flower has predicted, through network pharmacology and molecular docking analysis, that this compound may be one of eight key active ingredients responsible for the plant's anti-osteoporotic effects. It may exert this action by binding to targets such as MAPK1, MAPK3, RXRA, AKT1, ESR1, and STAT3, thereby

	regulating signalling pathways such as AGE-RAGE[27].

Note: Level 1: Metabolites in the sample match the database in MS1, MS2 and RT; Level 2: Metabolites in the sample match the database in both MS1 and MS2; Level 3: Metabolites in the sample match the database in MS1. Mass Error < 5 ppm.

Reference:

1. Wei, Y.; Yin, X.; Wu, H.; Zhao, M.; Huang, J.; Zhang, J.; Li, T.; Ning, J. Improving the flavor of summer green tea (Camellia sinensis L.) using the yellowing process. Food Chem. 2022, 388, 132982, doi:doi.org/10.1016/j.foodchem.2022.132982.
2. Yue, C.; Zeng, L.; Cao, H.; Zhang, S.; Hao, Z.; Wu, Z.; Luo, L. Aroma characteristics of Wuyi rock tea prepared from 16 different tea plant varieties. Food Chem. X 2023, 17, 100586. https://doi.org/10.1016/j.fochx.2023.100586.
3. Chen, G.; Zhu, G.; Xie, H.; Zhang, J.; Huang, J.; Liu, Z.; Wang, C. Characterization of the key differential aroma compounds in five dark teas from different geographical regions integrating GC–MS, ROAV and chemometrics approaches. Food Res. Int. 2024, 194, 114928, doi:doi.org/10.1016/j.foodres.2024.114928.
4. Guo, X.; Ho, C.-T.; Wan, X.; Zhu, H.; Liu, Q.; Wen, Z. Changes of volatile compounds and odor profiles in Wuyi rock tea during processing. Food Chem. 2021, 341, 128230. https://doi.org/10.1016/j.foodchem.2020.128230.
5. Lu, J.; Wu, Y.; Zhang, R.; Han, J.; Xiong, A.; Yu, Z. Change of the Volatile Compounds from Celery Leaves during Storage Based on HS-SPME-GC-MS and E-nose. Sci. Technol. Food Ind. 2024, 45, 212–222. https://doi.org/10.13386/j.issn1002-0306.2023040101.
6. Smith, J.A.; Maloney, D.J.; Clark, D.E.; Xu, Y.; Hecht, S.M.; Lannigan, D.A. Influence of rhamnose substituents on the potency of SL0101,an inhibitor of the Ser/Thr kinase, RSK. Bioorg. Med. Chem. 2006, 14, 6034–6042. https://doi.org/10.1016/j.bmc.2006.05.009.
7. Stathopoulou, K.; Schobesberger, S.; Bork, N.I.; Sprenger, J.U.; Perera, R.K.; Sotoud, H.; Geertz, B.; David, E.-P.; Christ, T.; Nikolaev, V.O.; et al. Divergent off-target effects of RSK N-terminal and C-terminal kinase inhibitors in cardiac myocytesDivergent off-target effects of RSK N-terminal and C-terminal kinase inhibitors in cardiac myocytes. Cell. Signal. 2019, 63, 109362. https://doi.org/10.1016/j.cellsig.2019.109362.
8. Sun, Y.; Tang, L.; Wu, C.; Wang, J.; Wang, C. RSK inhibitors as potential anticancer agents: Discovery, optimization, and challenges. Eur. J. Med. Chem 2023, 251, 115229. https://doi.org/10.1016/j.ejmech.2023.115229.
9. Xiang, L.; Feng, M.; Guo, X.; Li, Y.; Zhao, Z.; Liu, G.; Cai, S.; Xu, F. Studying the effects of Saposhnikoviae Radix on the phar-macokinetic profiles of 10 bioactive compounds originating from Astragali Radix in rat plasma by UHPLC-QTRAP-MS/MS. J. Ethnopharmacol, 2025, 337: 118813. https://doi.org/10.1016/j.jep.2024.118813
10. Ding, L.; Luo, C.; Weygant, N.; Chen, W.; Ru, D.; Lai, Y.; Wang, Y.; Li, H. Scutellarin suppresses ovarian cancer progression by targeting METTL5. Sci. Rep. 2025, 15, 18472. https://doi.org/10.1038/s41598-025-03411-y.
11. Yi, H.; Jiang, Y.; Li, W.; Shen, L.; Zhang, W.; Li, S.; Xu, Y.; Li, F. Scutellarin prevents obesity-induced renal fibrosisvia reduced activation of AP-1. J. Transl. Med. 2025, 23, 611. https://doi.org/10.1186/s12967-025-06616-x.

12. Sun, C.; Li, C.; Li, X.; Zhu, Y.; Su, Z.; Wang, X.; He, Q.; Zheng, G.; Feng, B. Scutellarin induces apoptosis and autophagy in NSCLC cells through ERK1/2 and AKT Signaling Pathways in vitro and in vivo. J. Cancer 2018, 9, 3247. https://doi.org/10.7150/jca.25921.
13. Zhang, X.; Ji, R.; Sun, H.; Peng, J.; Ma, X.; Wang, C.; Fu, Y.; Bao, L.; Jin, Y. Scutellarin ameliorates nonalcoholic fatty liver dis-ease through the PPARγ/PGC-1α-Nrf2 pathway. Free Radical Res, 2018, 52, 198–211. https://doi.org/10.1080/10715762.2017.1422602
14. Hu, C.; Tohge, T.; Chan, S.-A.; Song, Y.; Rao, J.; Cui, B.; Lin, H.; Wang, L.; Fernie, A. R.; Zhang, D.; et al. Identification of con-served and diverse metabolic shifts during rice grain development. Sci. Rep. 2016, 6, 20942. https://doi.org/10.1038/srep20942.
15. Jung, S.-H.; Kim, J.; Eum, J.; Choe, J.W.; Kim, H.H.; Kee, Y.; Lee, K. Velutin, an Aglycone Extracted from Korean Mistletoe, with Improved Inhibitory Activity against Melanin Biosynthesis. Molecules 2019, 24, 2549. https://doi.org/10.3390/molecules24142549.
16. Zhang, Y.; Xu, R.; Wang, K.; Li, X.; Lou, Y.; Cao, L.; Yang, W.; Qian, Y. Velutin Inhibits IL-1β-Induced Nucleus Pulposus Inflammatory and Extracellular Matrix Degradation Attenuating Mouse Intervertebral Disc Degeneration via the NF-κB and MAPK Pathways. Mediat. Inflamm. 2025, 2025, 9625485. https://doi.org/10.1155/mi/9625485.
17. Xie, C.; Kang, J.; Li, Z.; Schaussd, A.G.; Badger, T.M.; Nagarajan, S.; Wu, T.; Wu, X. The açaí flavonoid velutin is a potent anti-inflammatory agent: Blockade of LPS-mediated TNF-α and IL-6 production through inhibiting NF-κB activation and MAPK pathway. Nutr. Biochem. 2012, 23, 1184–1191. https://doi.org/10.1016/j.jnutbio.2011.06.013.
18. Guo, P.; Zeng, M.; Wang, S.; Cao, B.; Liu, M.; Zhang, Y.; Jia, J.; Zhang, Q.; Zhang, B.; Wang, R.; et al. Eriodictyol and Ho-moe-riodictyol Improve Memory Impairment in Aβ25–35-Induced Mice by Inhibiting the NLRP3 Inflammasome. Molecules, 2022, 27, 2488. https://doi.org/10.3390/molecules27082488.
19. Lieder, B.; Hoi, J. K.; Holik, A.-K.; Geissler, K.; Hans, J.; Friedl, B.; Liszt, K.; Krammer, G. E.; Ley, J. P.; Somoza, V. The flavanone homoeriodictyol increases SGLT-1-mediated glucose uptake but decreases serotonin release in differentiated Caco-2 Cells. PLoS ONE, 2017, 12(2), e0171580. https://doi.org/10.1371/journal.pone.0171580.
20. Xun, J.; Hu, Z.; Wang, M.; Jiang, X.; Liu, B.; Han, Y.; Gao, R.; Wu, X.; Zhang, A.; Yang, S.; et al. Hydroxygenkwanin suppresses peritoneal metastasis in colorectal cancer by modulating tumor-associated macrophages polarization. Chem.-Biol. Interact. 2024, 396, 111038. https://doi.org/10.1016/j.cbi.2024.111038.
21. Wollenweber, E.; Marx, D.; Favre-Bonvin, J.; Voirin, B.; Kaouadji, M. 3-methoxyflavones with unusual b-ring substitution from two species of notholaena. Phytochemistry 1988, 27, 2673–2676. https://doi.org/10.3390/molecules26123557
22. Anh Dinh, T. N.; Pham, T. L.; Nguyen, V. P.; Lee, T.; Min, B. S.; Kim, J. A. Polyphenols from the aerial parts of Lespedeza cune-ata and their inhibitory

effects on PTP1B and α-glucosidase: insights from in vitro and in silico analyses. J. Nat. Med. 2026. 1–15. https://doi.org/10.1007/s11418-026-02002-z.
23. Grijalva-Guiza, R. E.; Jiménez-Garduño, A. M.; Hernández, L. R. Potential Benefits of Flavonoids on the Progression of Ather-osclerosis by Their Effect on Vascular Smooth Muscle Excitability. Molecules, 2021, 26, 3557. https://doi.org/10.3390/molecules26123557.
24. Liao, F.; Li, S.; Wu, L.; Chen, J.; Luo, Z.; Zhong, M.; Li, Q.; Wang, W.; Li, G. Herbacetin Alleviates Influenza Virus-Induced Lung Injury and Fibrosis by Targeting the Neuraminidase Protein. Pharmaceuticals 2025, 18, 1306. https://doi.org/10.3390/ph18091306.
25. Bhutkar, M.; Verma, S.; Singh, V.; Kumar, P.; Tomar, S. Mechanistic Insights Into the Inhibition of Dengue Virus NS5 Methyl-transferase by Herbacetin. Proteins: Struct., Funct., Bioinf. 2025, 0, 1–9. https://doi.org/10.1002/prot.70108.
26. Qian, W.; Chen, X.; Gu, L.; Lin, X.; Chen, J.; Xu, D.; Wang, H.; Long, F.; Zhuge, Q.; Zhang, P.; et al. Herbacetin alleviates fer-roptosis via Hif-1α/SLC7A11/GPX4 axis in traumatic brain injury. Free Radical Biol. Med. 2025, 238, 179–193. https://doi.org/10.1016/j.freeradbiomed.2025.06.030.
27. Shen, N.; Li, Y.; Liu, Y.; Liu, Y.; Xin, H.; Cui, Y. Gypsophila oldhamiana leaves as a potential industrial resource of lipids, alkaloids, flavonoids and anti-osteoporosis components. Ind. Crops Prod. 2023, 196, 116510. https://doi.org/10.1016/j.indcrop.2023.116510.
